# RNA-Interference Pathways Display High Rates of Adaptive Protein Evolution in Multiple Invertebrates

**DOI:** 10.1534/genetics.117.300567

**Published:** 2018-02-01

**Authors:** William H. Palmer, Jarrod D. Hadfield, Darren J. Obbard

**Affiliations:** * Institute of Evolutionary Biology, University of Edinburgh, EH9 3FL, United Kingdom; † Centre for Infection, Evolution and Immunity, University of Edinburgh, EH9 3FL, United Kingdom

**Keywords:** adaptive evolution, antiviral immunity, population genetics, RNA interference, small RNA, transposable elements, viruses

## Abstract

Conflict between organisms can lead to a reciprocal adaptation that manifests as an increased evolutionary rate in genes mediating the conflict. This adaptive signature has been observed in RNA-interference (RNAi) pathway genes involved in the suppression of viruses and transposable elements in *Drosophila melanogaster*, suggesting that a subset of *Drosophila* RNAi genes may be locked in an arms race with these parasites. However, it is not known whether rapid evolution of RNAi genes is a general phenomenon across invertebrates, or which RNAi genes generally evolve adaptively. Here we use population genomic data from eight invertebrate species to infer rates of adaptive sequence evolution, and to test for past and ongoing selective sweeps in RNAi genes. We assess rates of adaptive protein evolution across species using a formal meta-analytic framework to combine data across species and by implementing a multispecies generalized linear mixed model of mutation counts. Across species, we find that RNAi genes display a greater rate of adaptive protein substitution than other genes, and that this is primarily mediated by positive selection acting on the genes most likely to defend against viruses and transposable elements. In contrast, evidence for recent selective sweeps is broadly spread across functional classes of RNAi genes and differs substantially among species. Finally, we identify genes that exhibit elevated adaptive evolution across the analyzed insect species, perhaps due to concurrent parasite-mediated arms races.

RNA-interference (RNAi) mechanisms include a diverse group of pathways, united by their use of Argonaute-family proteins complexed with short (20–30 nt) RNA molecules to target longer RNA molecules through sequence complementarity (Carmell *et al.* 2002; Meister 2013). These pathways regulate multiple biological processes that can be divided into three distinct subpathways in arthropods and nematodes, each represented by a characteristic class of small RNAs: the micro-RNA (miRNA), the short-interfering RNA (siRNA), and the piwi-interacting RNA (piRNA) pathways. The miRNA pathway processes endogenously encoded fold-back hairpins which, in their mature miRNA form, regulate gene expression and coordinate developmental processes (Alvarez-Garcia and Miska 2005; Chen *et al.* 2014; Ha and Kim 2014). The siRNA pathway has two distinct roles, depending on the endogenous or exogenous origin of its substrate. First, the endo-siRNA pathway processes endogenously encoded double-stranded RNA to regulate processes such as transposable element (TE) defense (Czech *et al.* 2008; Ghildiyal *et al.* 2008; Kawamura *et al.* 2008), chromosomal segregation (Hall *et al.* 2003; Huang *et al.* 2015), and heterochromatin formation (Deshpande *et al.* 2005). Second, the exo-siRNA (or viRNA) functions primarily as a form of antiviral immunity (Wang *et al.* 2006; Bronkhorst and van Rij 2014). The piRNA pathway forms a defense against TEs, which is germline limited in some species, and is mediated by piRNAs derived from endogenously encoded clusters of inactivated TE sequences and from active TEs (Klattenhoff and Theurkauf 2008; Thomson and Lin 2009; Czech and Hannon 2016; Lewis *et al.* 2018).

Within this framework, there is substantial mechanistic variation among species and RNAi-pathway components seem to be evolutionarily labile. For example, in nematodes, the mechanism and function of the piRNA pathway is not well conserved: primary piRNA-like small RNAs are encoded by short distinct loci instead of the clusters observed in flies and mammals, and they mediate the biogenesis of a separate endo-siRNA population transcribed by an RNA-dependent RNA polymerase (RdRP) and processed by Dicer (Duchaine *et al.* 2006; Das *et al.* 2008). Further, only one of the five major clades of nematode has retained Piwi-subfamily proteins—the canonical effector of the piRNA pathway—and instead rely solely on the (RdRP-produced) endo-siRNAs (Sarkies *et al.* 2015). The piRNA pathway can also take on entirely new roles, for example, duplications of *piwi* in *Aedes* mosquitoes has allowed the piRNA pathway to adopt an antiviral role in somatic tissues (Morazzani *et al.* 2012), while other *piwi* duplicates maintain the ancestral function (Miesen and Girardi 2015; Miesen *et al.* 2016).

The role of RNAi pathways in mediating intergenomic (host–virus) and intragenomic (host–TE, segregation distortion) (Ferree and Barbash 2007) conflict may make them a target of antagonistic host–parasite coevolution. This could result in balancing or directional selection on the loci in conflict, evidenced by the maintenance of polymorphism or elevated rates of adaptive fixation, respectively (*e.g.*, Anderson and May 1982; Ebert 2008). This has been well studied in *Drosophila* RNAi-pathway genes, which show elevated rates of adaptive protein evolution (Obbard *et al.* 2006, 2009), signatures of selective sweeps (Kolaczkowski *et al.* 2011; Obbard *et al.* 2011; Lewis *et al.* 2016), and sites with elevated protein evolution across the *Drosophila* phylogeny (Vermaak *et al.* 2005; Heger and Ponting 2007; Kolaczkowski *et al.* 2011). For example, a comparison of the antiviral RNAi genes *AGO2*, *Dcr-2*, and *r2d2* to their miRNA functional counterparts with no known role in conflict (the paralogs *AGO1*, *Dcr-1*, and *loqs*) shows a striking difference in rates of protein evolution, as well as a greater rate of adaptive amino acid substitution (Obbard *et al.* 2006). In addition, evolutionary rates of piRNA-pathway genes involved in transcriptional silencing are elevated and highly correlated with other piRNA-pathway genes across the *Drosophila* phylogeny (Blumenstiel *et al.* 2016).

Although some antiviral and anti-TE RNAi-pathway genes clearly display elevated rates of adaptive protein evolution in *Drosophila*, the generality of this pattern remains to be elucidated. Here we apply both traditional McDonald–Kreitman (MK) (McDonald and Kreitman 1991) and SnIPRE-style (Eilertson *et al.* 2012) analyses as well as selective sweep-based analyses (Nielsen *et al.* 2005; Pavlidis *et al.* 2013) to publicly available genome-scale data from three dipterans, two lepidopterans, a hymenopteran, and two clade V nematodes (Sarkies *et al.* 2015).

By combining estimates across species, we investigate the specific RNAi subpathways that may be the target of elevated positive selection. This allows us to estimate the rates of adaptation across species, thereby improving single-gene estimates and allowing us to identify genes that are undergoing parallel adaptation across the taxa analyzed. Finally, we summarize the evidence for recently completed and ongoing selective sweeps in RNAi genes across these eight taxa. We conclude that rapid evolution of RNAi genes is a general phenomenon in these eight invertebrates, although evidence for recent sweeps is highly contingent on the focal species.

## Materials and Methods

### Selection of genes for analysis

Putative RNAi-pathway genes of *Drosophila melanogaster* and *Caenorhabditis elegans* were used to find homologs in six insects and two nematode species (Supplemental Material, Table S1 and Table S2). For the six insect species, we classified these genes as miRNA, piRNA, siRNA, or viRNA based on a literature search (Table S1). Where a gene was implicated in more than one subpathway, we assigned it to the pathway that has been independently experimentally validated most often. Although the viRNA and siRNA pathways are not easily separable, we make this distinction based on the hypothesis that these genes may be evolving adaptively in response to viruses, as these genes have direct experimental evidence of an antiviral role in *D. melanogaster*. We also split the piRNA-pathway genes among three functional categories: post-transcriptional silencing effectors, transcriptional silencing effectors, and biogenesis machinery. A gene was considered a biogenesis factor if piRNA levels decrease upon loss of function, an effector if piRNA-pathway function is compromised without reducing piRNA levels, and a transcriptional silencing effector if the effector is involved in transcriptional silencing (Table S1). Finally, we selected 65 piRNA genes in *D. melanogaster* with known tissue specificity to calculate rates of adaptation in the germline *vs.* the somatic follicle cells (Table S3). This gene list contains the core of the piRNA-pathway genes independently validated in two of three screens for piRNA-pathway constituents (Czech *et al.* 2013; Handler *et al.* 2013; Muerdter *et al.* 2013).

Homologs of the *D. melanogaster* and *C. elegans* genes were identified using a two-step process. First, a hidden Markov model (HMMer) (Eddy 2008) was used to find the best reciprocal best hits for a gene of interest using predicted protein sets (if available) or UniProtKB. If no hit was found, then Exonerate was used to identify unannotated homologs in the genome using the model “protein2genome” (Slater and Birney 2005). If Exonerate was unable to model a homolog then this gene was classified as missing, either due to gene loss or an incomplete genome assembly. We defined genes as duplicates (paralogs) if multiple regions of a genome shared a best hit to a reference gene, and if these regions showed substantial sequence divergence between them (*i.e.*, they were not obviously a misassembly duplicate or allelic). Because the large divergence times between insects and nematodes and the complexity of RNAi pathways in nematodes make homology assignment uncertain, we restricted our gene-level analyses to insects.

### Population genomic data

We used previously published population genomic data for *D. melanogaster* (Lack *et al.* 2015), *D. pseudoobscura* (Pseudobase) (McGaugh *et al.* 2012), *Anopheles gambiae* (The *Anopheles gambiae* 1000 Genomes Consortium 2014), *Heliconius melpomene* (Kronforst *et al.* 2013), *Bombyx mandarina* (Xia *et al.* 2009), *Apis mellifera* (Harpur *et al.* 2014), *Pristionchus pacificus* (Rödelsperger *et al.* 2014), and *C. briggsae* (Thomas *et al.* 2015) for our analyses (Table S4). For both *Drosophila* species, we used previously published haplotype data (haploid sequencing of *D. melanogaster* and inbred lines of *D. pseudoobscura*). For the other taxa, we obtained raw sequencing reads from the European Bioinformatics Institute European Nucleotide Archive (identifiers provided in Table S4) and mapped them to the most recent reference genome for each species using Bowtie2 (Langmead and Salzberg 2012) with default settings. We used GATK’s HaplotypeCaller on each individual separately (DePristo *et al.* 2011) to call variants in a 200-kb region surrounding each gene of interest (*i.e.*, 100 kb either side of the RNAi gene, unless the contig was <200 kb). For high coverage data sets (*A. mellifera*, *H. melpomene*, *C. briggsae*, *A. gambiae*, and *P. pacificus*) we excluded sites with a read depth lower than five, but we reduced this threshold to two for the low-coverage *B. mandarina*. All sites above this filter were included in the analysis. After mapping and filtering sites we created two randomly resolved pseudohaplotype sequences per individual (*i.e.*, without any phase information) from the sites that remained, and these were used for downstream analyses (none of which depend on phase). Only one haplotype was sampled from each *C. briggsae* and *P. pacificus* individual, as the sequenced individuals were reported to be highly homozygous. In *H. melpomene*, we occasionally observed long stretches of high divergence shared by multiple individuals. We assumed these to be possible cases of either contamination, inversions that have recently risen to a high frequency, or introgression (Pardo-Diaz *et al.* 2012), and we removed these haplotypes.

To calculate divergence between genes we used the out-group species *D. simulans*, *D. miranda*, *H. hecale*, *B. huttoni*, *A. christyi*, and *A. melas*; and to polarize mutations for sweep analyses we used *A. cerana*, *C. nigoni*, and *P. exspectatus* (Table S4). Out-groups were selected based on their divergence from the in-group species (∼1–10% divergence of all sites) and on the availability of genomic data. For *A. gambiae* we tested out-groups with low (*A. melas*) and high (*A. christyi*) divergence times, as most *Anopheles* species are too close or too divergent to provide a robust out-group for MK tests (Obbard *et al.* 2007), and our results remain qualitatively the same for both out-groups (*A. melas* used for the presented analyses). For *D. simulans* (FlyBase, r2.02), *D. miranda* (Pseudobase, MSH22 strain), *A. melas* (VectorBase, CM1001059 strain, AmelC1 assembly), *A. christyi* (VectorBase, ACHKN1017 strain, AchrA1 assembly), *B. huttoni* (Sackton *et al.* 2014) (BioProject PRJNA198873), and *P. exspectatus* (WormBase, Bioproject PRJEB6009), the out-group reference assemblies were publicly available and used as provided. However, the *C. nigoni* reference assembly sequence is contaminated with the more divergent nematode *C. afra* (Thomas *et al.* 2015), and *C. nigoni* is the only currently suitable out-group for *C. briggsae*. We therefore applied a sliding window across the alignments between *C. nigoni* and *C. afra*, and arbitrarily excluded regions that were >6 SD from the mean divergence. Published reference assemblies were not available for *A. cerana* and *H. hecale*. To generate out-group sequences for these species, we iteratively remapped reads (*H. hecale*: ERR260306; A. cerana: SRR957079) to the respective *A. mellifera* and *H. melpomene* references, each time updating the previous reference with homozygous nonreference calls. These reads were mapped with Bowtie2 and then remapped with the divergent alignment software Stampy (Lunter and Goodson 2011). Homozygous nonreference calls (enriched for sites divergent between the in-group and out-group) were made with GATK’s HaplotypeCaller, with the heterozygosity parameter set to the expected divergence between species. Such sequences will not perfectly reflect the true out-group sequence and they are expected to be biased toward the in-group, downwardly biasing estimates of divergence in high-divergence regions. However, we confirmed that this approach works well by iteratively mapping *D. simulans* to *D. melanogaster* and then comparing the result with the known *D. simulans* assemblies (*K*_S_ = 0.10 for iterative mapping *vs.* *K*_S_ = 0.12 for the true assembly). While bias probably remains, it is unlikely to spuriously elevate the inferred rates of one class of genes relative to the other. More generally, our approach to mapping, filtering, and variant calling may be prone to such biases, but they are unlikely to differentially affect gene classes of different function.

For the MK-based analyses, target sequences were aligned as amino acids using MUSCLE (Edgar 2004) and then each gene alignment was examined manually to remove putative misalignments. Likely misalignments were identified by eye as regions of unusually high divergence with no amino acid similarity to the consensus sequence, often occurring at the ends of the gene. We assumed these were caused by misassembly and removed these blocks from the alignment. Within-species data were aligned first, and then a consensus sequence of this alignment was used to align against the out-group sequence. Synonymous and nonsynonymous substitutions between species were inferred using codeml from the PAML package using the YN00 model (Yang and Nielsen 2000), which estimates substitution rates using an approximation to maximum likelihood methods, while accounting for base composition differences between codon positions and differences in transition/transversion rates.

### Rates of adaptive protein evolution by pathway

To estimate the rate of adaptive protein evolution in different functional classes of gene, and to test for differences in rate between classes, we used two different approaches derived from the MK test (“MK framework”) (McDonald and Kreitman 1991). The MK framework combines polymorphism and divergence data from putatively unconstrained (synonymous) and potentially selected (nonsynonymous) variants to infer an excess of nonsynonymous fixations that can be attributed to positive selection. This framework was later formalized in several maximum likelihood and Bayesian methods to estimate α, the proportion of nonsynonymous substitutions that are adaptive (Charlesworth 1994; Fay *et al.* 2001, 2002; Smith and Eyre-Walker 2002; Sawyer *et al.* 2003; Bierne and Eyre-Walker 2004; Welch 2006). However, α and related statistics can be biased by slightly deleterious mutations. This is because such mutations are unlikely to fix but do contribute substantially to polymorphism (McDonald and Kreitman 1991; Eyre-Walker 2002; Charlesworth and Eyre-Walker 2008; Eyre-Walker and Keightley 2009; Gossmann *et al.* 2012). We used DFE-alpha and SnIPRE to estimate rates of adaptive evolution. These complementary approaches model the population-genetic processes responsible for these biases (DFE-alpha) or the resulting genome-wide variability caused by these biases (SnIPRE) (Eyre-Walker and Keightley 2009; Eilertson *et al.* 2012).

In the first approach, we used an explicit population-genetic model to estimate the number of adaptive nonsynonymous substitutions per site (DFE-alpha; Eyre-Walker and Keightley 2009). This approach has the advantage that it provides direct estimates of the parameters of interest and it explicitly models changes in population size [as reflected by the site frequency spectrum (SFS) of unconstrained sites] and the distribution of deleterious fitness effects, which might otherwise bias estimates (Keightley and Eyre-Walker 2007; Eyre-Walker and Keightley 2009). However, as currently implemented, this method does not allow data to be directly combined among species. Therefore, to obtain more precise homolog- and pathway-based estimates, we combined per-gene point estimates from DFE-alpha using a linear mixed model (including their estimated uncertainty; *i.e.*, a meta-analysis; see Text S1 in File S1). In the second approach, we used an extension of the SnIPRE model (Eilertson *et al.* 2012), which reframes the MK framework as a linear model in which polymorphism and substitution counts are predicted by synonymous or nonsynonymous state. Although this model does not explicitly consider the same underlying population-genetic processes, it does permit a straightforward extension to natively include gene, homolog, pathway, and host species as predictors, and therefore provides a direct test of the questions of interest (although at a cost of potentially less accurate or arbitrarily scaled parameter estimates; see Text S1 in File S1). We have reimplemented the SnIPRE model using the Bayesian generalized linear mixed modeling R package MCMCglmm (Hadfield 2010). A detailed description of the study design and analytical and statistical methods is provided in Text S1 in File S1, along with annotated R code necessary to fit the described models.

### Selective sweep analysis

The recent spread of a positively selected allele leaves characteristic patterns of diversity and allele frequencies in the genomic region surrounding the selected site, and these can be used to detect recent adaptive substitutions (*e.g.*, Smith and Haigh 1974; Barton 1998; Nielsen *et al.* 2005). We used SweeD (Pavlidis *et al.* 2013; derived from Sweepfinder, Nielsen *et al.* 2005) to search for evidence of recent selective sweeps in the regions surrounding RNAi genes. The algorithm scans the genome and at a user-defined interval calculates the composite likelihood of the observed SFS under a model of a selective sweep centered on that site *vs.* a standard neutral model. The ratio of the two composite likelihoods (CLR) is then used as a test statistic, with significance assessed by coalescent simulation (see Figure S1 in File S2 and Text S2 in File S1). We used this method to scan 200 kb (or less if the reference genome contig was <200 kb) surrounding each gene of interest in each species. For each focal region, we polarized the SFS by parsimony between the out-group reference genome and the in-group consensus sequence, which we aligned with LastZ ungapped alignment (Harris 2007). This simple parsimony-based inference of ancestral states risks mispolarization of low frequency polymorphism as high frequency-derived alleles; however, we assume this does not differentially affect control and RNAi genes. We did not assume an ancestral state for fixed differences that were invariant in our in-group (*i.e.*, these sites were folded). This will make the analysis more robust to possible errors during contig alignment, because misalignment would manifest itself as regions of increased divergence between species. We included invariant sites in the analysis as a characteristic signature of a recent sweep is a lack of diversity; so including invariant sites in Sweepfinder analyses can greatly improve statistical power (Nielsen *et al.* 2005). This comes with a risk of increased false positives (Huber *et al.* 2016), but including these sites should not differentially affect RNAi and control genes unless there is a consistent difference in neutral mutation rates or depth of coverage between these two classes of genes. We have confirmed that there are no consistent differences in read depth between control and RNAi genes. The SweeD analysis provides CLR values for equidistant points across the genome, with CLR values forming a “peak” in areas with high support for a sweep. To assess whether RNAi genes have experienced more sweeps than control genes in six of our eight species (*B. mandarina* and *P. pacificus* were not tested because the published genome assemblies are unannotated), we counted the number of RNAi and control genes that overlapped significant peaks in the CLR statistic (based on the significance threshold provided by coalescent simulation; Figure S1 in File S2 and Text S2 in File S1). If consecutive peaks occurred within 1 kb of each other, we classified them as a single broad peak, such that the contig was split into “sweep-positive” and “sweep-negative” areas. We then classified all genes along the contig as to whether they overlapped a sweep-positive area or not, and whether or not they were an RNAi gene. We used a binomial test to assess whether RNAi or control classes had more sweep-positive genes than expected given the summed gene length for each class.

To test whether sweeps were enriched in any particular subpathway, we normalized the maximum CLR statistic in a gene by the expected significance threshold from coalescent simulations and modeled these values ($\tilde{CLR}$) using the following linear mixed model:

$${\tilde{CLR}}_{klm}=\;\beta_{0}+\beta_{\text{Class:}l}+u_{\text{Organism:m}}+\varepsilon_{klm}.$$

Here, $\beta_{\text{Class:}l}$ is a fixed effect for the pathway each gene is assigned (miRNA, siRNA, piRNA, or viRNA), $u_{\text{Organism:m}}$ is a random effect for species *m*, and $\varepsilon_{klm}$ is the error term.

In the four organisms for which we have haplotype information (*D. melanogaster*, *D. pseudoobscura*, *P. pacificus*, and *C. briggsae*), we additionally tested for ongoing or soft sweeps using the haplotype-based nSL statistic (Ferrer-Admetlla *et al.* 2014). The nSL statistic is similar to the more widely used iHS statistic (Voight *et al.* 2006) except that distance is measured in polymorphic sites rather than the genetic map distance (Ferrer-Admetlla *et al.* 2014). This genome scan calculates the average number of consecutive polymorphisms associated with either the ancestral or derived allele at each polymorphic site along the contig across all pairwise comparisons. Areas with long-range linkage disequilibrium will therefore be identified through SNPs with extreme nSL values.

### Data availability

The data used to fit the DFE-alpha meta-analysis and the cross-species SnIPRE analysis are provided on Figshare (Data S1 and S2 in File S1; DOI: 10.6084/m9.figshare.5843991.v1). For DFE-alpha data, each line corresponds to a gene within a species, where we include the output of DFE-alpha, the bootstrap SE, and associated RNAi pathways. For the SnIPRE data, each line corresponds to a type of mutation in a gene in a species—such that there are four lines per gene–species combination, and includes associated RNAi pathways and the number of nonsynonymous and synonymous sites (*L*_N_ and *L*_S_). Gene alignments are available via Figshare (DOI: 10.6084/m9.figshare.5621455).

## Results

### Evidence of genome-wide adaptive substitution in insects, but not nematodes

**Figure 1:**
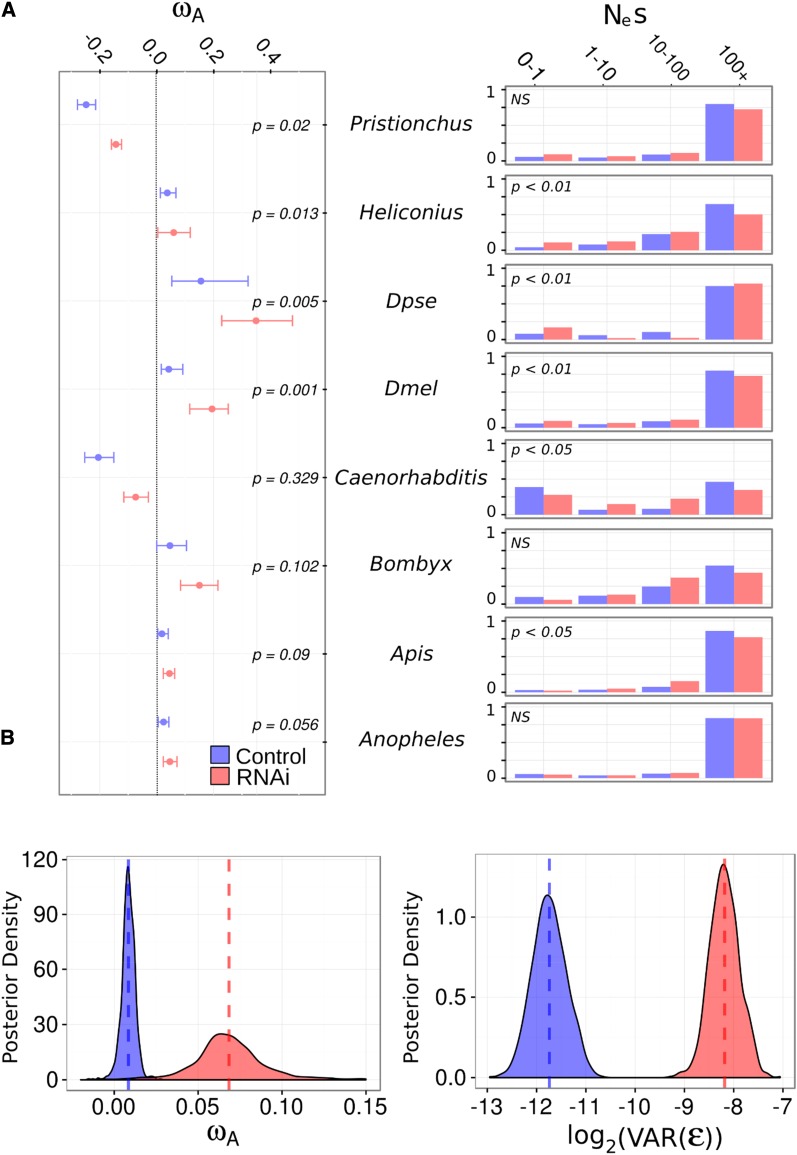
ω_A_ and the DFE differ between RNAi genes and other genes. (A) Left: For each species, ω_A_ estimates and 95% bootstrap confidence intervals are plotted for control (*i.e.*, non-RNAi; blue) and RNAi (red) genes. Significance was determined by permutation. Right: The estimated discretized DFE for each species, with the proportion of mutation with deleterious *N*_e_*s* values in each category given for non-RNAi (blue) and RNAi (red) genes. (B) The posterior distribution of estimated ω_A_ for RNAi (red) *vs.* control (blue) genes, showing that RNAi genes have much greater ω_A_ estimates (left) and greater residual gene-level variation (right), indicating RNAi genes display higher rates of adaptive amino acid substitution but are more variable.

Position-matched “control” genes (lacking RNAi-related function) allowed us to estimate the average genome-wide rate of adaptation, assuming that proximity to RNAi gene has no effect on their rate of adaptive evolution. Our findings broadly agree with previous analyses; suggesting a substantial fraction of amino acid substitution is adaptive across insect species (Figure 1). All insect species shared similar estimates (ω_A_ from 0.02 to 0.05) except for *D. pseudoobscura*, which exhibited an extremely high ω_A_ value of 0.16 adaptive nonsynonymous substitutions per synonymous substitution per site with a 95% bootstrap interval [0.05, 0.32]. Although we only sampled two nematode lineages, it is notable that both ω_A_ estimates were negative (*C. briggsae*: −0.20 [−0.25, −0.15]; *P. pacificus:* −0.24 [−0.27, −0.21]). This is consistent with the previously noted high ratio of nonsynonymous to synonymous polymorphism (π_A_/π_S_) ratio in these species, and perhaps suggests population structure and local adaptation (Rödelsperger *et al.* 2014; Thomas *et al.* 2015). We also calculated α, or the proportion of adaptive substitutions for each species, which reflects the same patterns observed for ω_A_ (Figure S2 in File S2).

The cross-species SnIPRE-like model provides a formal comparison of adaptive divergence in the insect species. The structure of the model forces comparison relative to one species, for which we chose *D. melanogaster*. *A. gambiae* and *B. mandarina* had levels of putatively adaptive nonsynonymous divergence that were indistinguishable from those of *D. melanogaster* (MCMCp = 0.489 and MCMCp = 0.616, respectively). Consistent with the DFE-alpha estimates of ω_A,_ *A. mellifera* and *H. melpomene* had significantly less adaptive nonsynonymous divergence than *D. melanogaster* (MCMCp = 0.04 and MCMCp < 3 × 10^−4^, respectively); whereas *D. pseudoobscura* had an increased excess of nonsynonymous divergence (MCMCp = 0.0005). Other species-specific SnIPRE parameters can be found in Text S1 in File S1.

### RNAi genes consistently display more adaptive protein substitution than other genes

For each focal species, we estimated the distribution of fitness effects of new mutations using DFE-alpha for RNAi-pathway and non-RNAi (control) genes. We fitted two models, one in which RNAi and control genes shared a single distribution of fitness effect (DFE), and the second in which each class of gene had a separate DFE. We then compared these models using a likelihood ratio test. In *D. melanogaster*, *D. pseudoobscura*, *H. melpomene*, *A. mellifera*, and *C. briggsae*, models in which control and RNAi genes have separate DFE parameters fitted the data significantly better than a model in which the two classes share a single DFE (Figure 1). Although there is no clear or universal trend, the DFE of control genes generally seemed slightly shifted toward more deleterious mutations than RNAi genes. For example, in most lineages (not *D. pseudoobscura* or *A. gambiae*), the estimated DFE had a higher proportion of strongly deleterious mutations in control genes than RNAi genes, which suggests less constraint in RNAi genes. However, the overall shape of the DFE is quite different between species. This either indicates that in these species gene function may play a smaller role than other factors in patterns of polymorphism, such as the effective population size, or that the DFE is estimated with low precision.

We then compared rates of adaptive amino acid substitution in RNAi genes to those in the non-RNAi control genes in each lineage by pooling polymorphism and divergence data for the two classes as input to DFE-alpha (Figure 1). In every species tested, the point estimate of class-wide ω_A_ was greater in RNAi genes than control genes. Although the effect was often small, the difference was individually significant in *D. melanogaster*, *D. pseudoobscura*, *H. melpomene*, and *P. pacificus*. To quantify the overall difference, we analyzed individual gene estimates of ω_A_ in a linear mixed model framework (*i.e.*, a meta-analysis) to estimate cross-species rates of adaptive evolution in control and RNAi genes (Model 1 in Text S1 in File S1 and Figure 1). We found the cross-species ω_A_ was significantly greater for RNAi genes than control genes, estimated as ω_A_ = 0.062 [0.049, 0.078] *vs.* ω_A_ = 0.01 [0.0009, 0.019] (*P* < 0.001). In addition, the residual gene-level variance was also much greater (MCMCp < 0.001) for RNAi genes (0.0037, [0.0022, 0.0051]) than control genes (0.0003, [0.0001, 0.0004]), implying that ω_A_ is more variable in this class than among genes in general and consistent with a subset of RNAi genes or pathways undergoing extreme rates of adaptive amino acid substitution (Figure 1). However, the coefficient of variation was not significantly different between RNAi and control genes, indicating these differences in residual variances are consistent with a mean–variance relationship in the rates of RNAi-pathway genes (Figure S4 in File S2).

### Adaptive rates are high in piRNA and viRNA pathways

**Figure 2:**
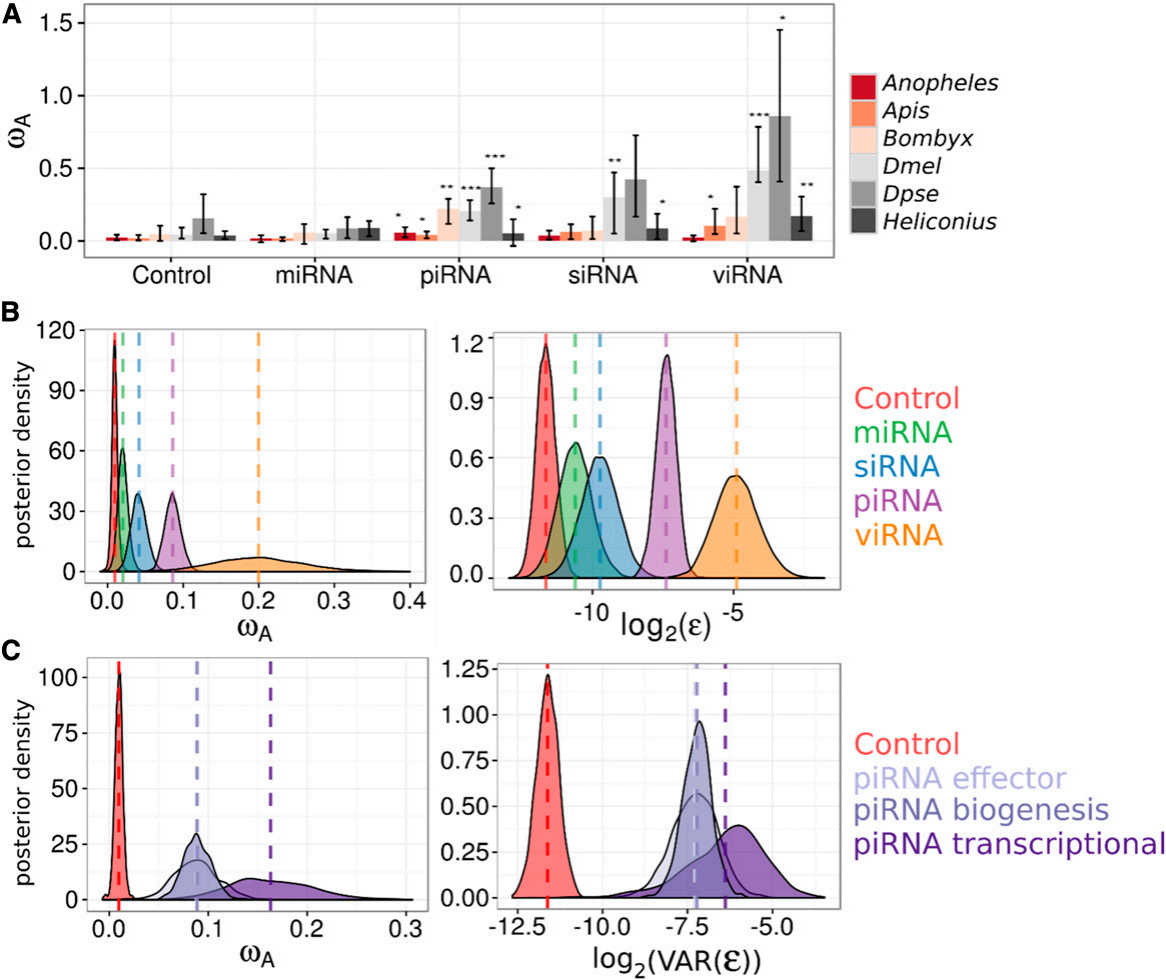
DFE-alpha estimates of ω_A_ differ among RNAi subpathways. (A) ω_A_ estimates from pooled polymorphism and divergence data across insect RNAi subpathways using DFE-alpha. The ω_A_ statistic was estimated for each subpathway in each organism and confidence intervals obtained by bootstrapping across genes. Significance was assessed by permutation tests between subpathway and control genes for each organism. * *P* < 0.05, ** *P* < 0.01, *** *P* < 0.001. (B) Individual gene DFE-alpha ω_A_ estimates were analyzed using a linear mixed model in MCMCglmm (see Text S1 in File S1) and show that (left) the viRNA pathway exhibits the fastest rate of adaptive protein substitution, followed by the piRNA pathway, and that among-gene variance shows the same pattern (right). (C) Individual gene DFE-alpha ω_A_ estimates were analyzed in MCMCglmm, except that the piRNA pathway was further split into genes involved in transcriptional silencing, piRNA biogenesis, or piRNA-mediated effectors of silencing. The posterior distributions of these three effect sizes *vs.* control genes are plotted. All three piRNA functions are targets of elevated positive selection and have large residual variances, although genes mediating transcriptional silencing have greater point estimates for both.

The higher rate of adaptive substitution seen in RNAi genes as a whole could result from slightly elevated positive selection across all components, or to a subset of the genes or pathway being substantially elevated. The higher gene-level variance seen in RNAi genes (above) suggests the latter, and to test this we pooled polymorphism and divergence data by subpathway for each insect species to calculate rates of adaptation in miRNA, siRNA, viRNA (*i.e.*, confirmed antiviral siRNA in *D. melanogaster*), and piRNA pathways (Figure 2). In each species, the piRNA pathway exhibited a significantly greater rate of adaptive amino acid substitution than control genes, and miRNA-pathway genes showed similar rates to control genes. Rates of adaptation for the siRNA (both endo-siRNA and viRNA) pathway were greater in only a subset of lineages. The magnitude of rates and proportion of lineages nominally significant in the test increased upon removing endo-siRNA genes and restricting the analysis to viRNA genes only. For all subsequent analyses, we analyzed these pathways separately to test the hypothesis that the core antiviral RNAi genes have elevated rates of adaptive evolution.

To formalize the effect of pathway (miRNA, piRNA, nonantiviral endo-siRNA, or viRNA) while accounting for variability in adaptation across species (Model 2 in Text S1 in File S1 and Figure 2), we performed a meta-analysis of ω_A_ estimates in individual genes from DFE-alpha, fitting pathway as a fixed effect. The piRNA, viRNA, and endo-siRNA pathways were each significantly different from control genes (control ω_A_ =0.01 [0.002, 0.018]; piRNA MCMCp < 0.001; viRNA MCMCp = 0.002; siRNA MCMCp = 0.004; for MCMCp value calculation, see the Text S1 in File S1), with cross-species estimates of ω_A_ of 0.08 [0.06, 0.10], 0.18 [0.06, 0.30], and 0.03 [0.01, 0.05], respectively. The viRNA-pathway ω_A_ estimate was not significantly greater than the piRNA pathway (MCMCp = 0.07), but was greater than the endo-siRNA pathway (MCMCp = 0.01), and the miRNA pathway (MCMCp < 0.001). The ω_A_ estimate for the piRNA pathway was significantly greater than the endo-siRNA (MCMCp = 0.002) and the miRNA pathways (MCMCp < 0.001). Consistent with our analysis of pooled polymorphism and divergence data, the rate of adaptive evolution in the miRNA pathway (ω_A_ = 0.01 [−0.001, 0.02]; MCMCp = 0.09) was not significantly different from control genes. Our linear models included pathway-specific error variances, which were lower for control genes (3 [2, 4] × 10^−4^) and miRNA-pathway genes (7 [2, 12] × 10^−4^) than for endo-siRNA (13 [4, 22] × 10^−4^), piRNA (66 [37, 97] × 10^−4^), and viRNA-pathway genes (0.04 [0.007, 0.86]); consistent with a great variation in adaptive rates in these pathways. As in the comparison between RNAi and control genes, these elevated variances in piRNA, siRNA, and viRNA pathways could be explained by the elevated mean rates in these pathways (Figure S4 in File S2).

**Figure 3:**
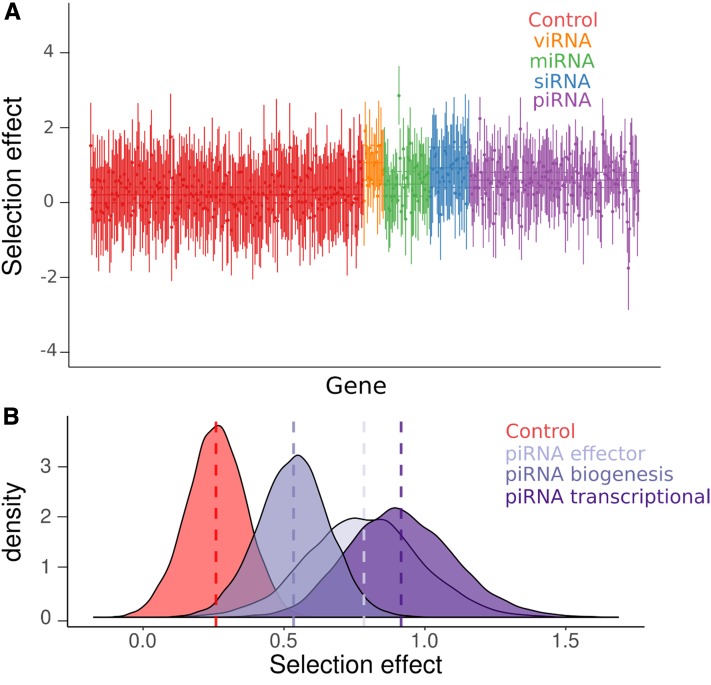
SnIPRE-like selection effects. (A) SnIPRE selection effect with 95% confidence intervals (species-level effects removed) are plotted for each gene in each species, colored according to the gene’s role in the RNAi pathway. Solid horizontal lines signify the mean selection effect for each RNAi subpathway (or control genes) with dotted lines signifying the 95% confidence intervals for the subpathway mean. SnIPRE and DFE-alpha analyses are consistent in suggesting that the viRNA, endo-siRNA, and piRNA pathways have more adaptive amino acid substitutions than control genes. The largest selection effect was seen in the *B. mandarina Dcr-1* locus, with a selection effect of 2.95 (Figure S6 in File S2). (B) We also performed a SnIPRE analysis after dividing the piRNA pathway into three functional classes, as in Figure 2. The posterior distributions of selection effects associated with each piRNA function are plotted. Similar to DFE-alpha, SnIPRE identifies all three pathways as significantly elevated relative to control genes; however, in the SnIPRE analysis, transcriptional silencing genes have a significantly greater adaptive rate than biogenesis factors.

We repeated the subpathway-level analysis using a SnIPRE-like model (Eilertson *et al.* 2012) to estimate the average selection effect within subpathways across organisms, without making any explicit assumptions about the DFE. Although SnIPRE can be used to provide estimates of population-genetic parameters, we limit our discussion to the “selection effect” statistic, where negative values are consistent with constraint and positive values with adaptive protein evolution, and magnitude reflects the strength of positive or negative selection. Consistent with our analysis of DFE-alpha estimates, the SnIPRE model identified a mean positive selective effect estimated across species (selective effect = 0.25 [0.02, 0.46], MCMCp = 0.03), with large variance among genes (Figure 3). Again, viRNA, endo-siRNA, and piRNA pathway-level selection effects were significantly elevated compared to control genes (viRNA: 1.10 [0.63, 1.57], MCMCp < 5 × 10^−4^; nonantiviral siRNA: 0.96 [0.44, 1.52], MCMCp = 0.02; piRNA: 0.63 [0.44, 0.84], MCMCp < 3 × 10^−4^); with the viRNA pathway exhibiting a significantly larger effect than the piRNA (MCMCp = 0.006), but not the endo-siRNA (MCMCp = 0.66). In agreement with the DFE-alpha analysis, the miRNA pathway was not significantly different from control genes (MCMCp = 0.07) and had a selection effect of 0.53 [0.20, 0.86].

### Adaptation is elevated in all major piRNA-pathway functions, but is most enriched in transcriptional silencing

Rapid adaptation in *Drosophila* piRNA-pathway genes has been hypothesized to be the result of fluctuating selection for increased TE defense and decreased off-target genic silencing (Blumenstiel *et al.* 2016). A prediction of this hypothesis is that genes involved in transcriptional silencing would be under increased positive selection. We tested this prediction by further dividing the piRNA pathway into effectors (*e.g.*, PIWIs), biogenesis factors (*e.g.*, adapter proteins), and transcriptional silencing factors, and then using single-gene polymorphism and divergence data to estimate ω_A_ and the selection effect for each piRNA functional category (Model 3 in Text S1 in File S1). We found all piRNA functional groups are significantly greater than control genes (MCMCp < 0.001) (Figure 2C), and that transcriptional silencing genes (ω_A_ = 0.16 [0.08–0.25]) have greater adaptive rates than effectors (MCMCp = 0.04, ω_A_ = 0.08 [0.04–0.13]) and biogenesis factors (MCMCp = 0.03, ω_A_ = 0.08 [0.05–0.11]). This result holds when excluding *Drosophila* transcriptional silencing factors *rhino*, *deadlock*, and *cutoff*, which are products of recent gene duplication or *de novo* formation (Figure S3 in File S2), and may not have evolutionary rates that are directly comparable to other genes.

We also estimated the average selection effect for each functional process of the piRNA pathway using the SnIPRE approach. Similar to the DFE-alpha meta-analysis, we find that all piRNA functional categories have elevated positive selection relative to control genes (biogenesis: MCMCp = 0.018; effector: MCMCp = 0.012; transcriptional silencing: MCMCp = 0.0004), that transcriptional silencing factors had the largest average selection effect of 0.92 [0.58, 1.31], and that genes involved in transcriptional silencing were significantly greater than biogenesis factors (selection effect: 0.53 [0.29, 0.78], MCMCp = 0.027) (Figure 3B). In contrast to the DFE-alpha meta-analysis, however, genes involved in transcriptional silencing were not significantly greater than effector genes (0.78 [0.40, 1.19], MCMCp = 0.68), and pathway-level point estimates of these selection effects were much closer (Figure 2C and Figure 3B).

### Individual genes in the piRNA and viRNA pathway show elevated adaptation

**Figure 4:**
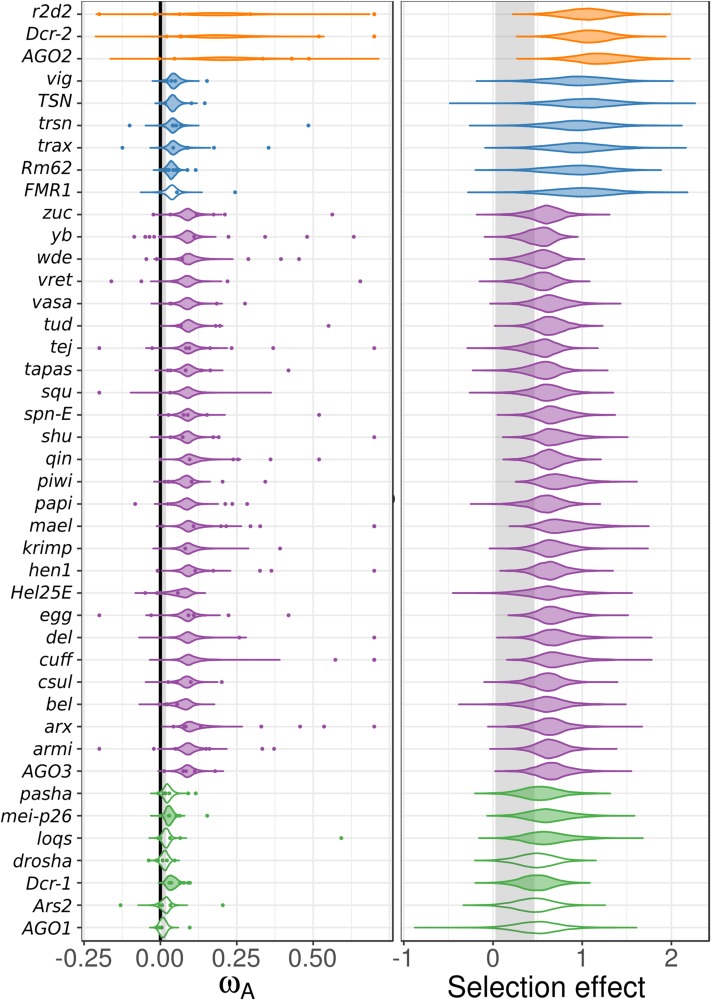
Cross-species, homolog-level estimates of ω_A_ and selection effects. (Left) Individual homolog ω_A_ estimates (colored points) were calculated using DFE-alpha and analyzed using a linear mixed model with subpathway as fixed effect and species and homolog as a random effect (estimate uncertainty was included by incorporating bootstrap intervals as measurement error variance). The posterior distributions of the cross-species estimate for ω_A_ for each homolog are plotted, and shaded if significantly different from the control gene distribution (region shaded gray). Single-gene estimates of ω_A_ > 0.75 are plotted at 0.75 for clarity. (Right) The analogous analysis performed using SnIPRE, with the posterior distribution of homolog-level selection effects plotted. Both analyses find little variation among homologs after accounting for subpathway, and homolog-level analyses generally mirror pathway-specific analyses. See Figure S4 in File S2 for the equivalent models that exclude the fixed effect of pathway.

The higher overall rates of adaptive protein substitution seen in RNAi genes may result from the engagement of some genes in an evolutionary arms race (*e.g.*, with viral suppressors of RNAi), a response to the selection imposed by the invasion of novel parasites (*e.g.*, TEs), or a trade-off between the specificity and sensitivity of genome defense (Obbard *et al.* 2006; Aravin *et al.* 2007; Blumenstiel *et al.* 2016). We used a linear mixed model to combine single-gene estimates of ω_A_ from DFE-alpha across multiple species to identify candidate arms-race genes in the RNAi pathways, fitting subpathway as a fixed effect with homolog and organism as random effects and subpathway-specific error variances. We found little variation among genes in a subpathway after accounting for subpathway, and in most cases there was not enough information to differentiate individual genes from the subpathway mean (Figure 4, left). Consequently, all genes in the rapidly evolving viRNA and piRNA subpathways were identified as having significantly greater adaptive rates than control genes. Most (five of six) siRNA-pathway genes, and two of seven miRNA-pathway genes were also identified as having significantly elevated adaptive rates. Although a model that accounts for pathway is statistically preferable if pathways are meaningful, any errors in assigning pathway “membership” would introduce bias to the estimates for misclassified genes. We therefore also estimated homolog-specific effects in a model that excludes the subpathway effect (Model 2B in Text S1 in File S1). This model finds significant evidence for positive selection in fewer genes (Figure S5A in File S2), including 13 of 22 piRNA genes, 2 of 3 viRNA genes, and no genes in the siRNA or miRNA pathway.

We also performed this homolog-level analyses using the SnIPRE approach. Similar to the DFE-alpha meta-analysis, we found very little information after accounting for subpathway (Figure 4, right), resulting in low among-gene variation within RNAi subpathways. When we excluded subpathway effects, we found a similar result to the homolog-level DFE-alpha meta-analysis without subpathway, except fewer piRNA-pathway genes are nominally significant (6 of 22 genes) (Figure S5B in File S2). Notably, *maelstrom*, *eggless*, *piwi* (incorporating the dipteran duplicate *aub*), *AGO2*, and *Dcr-2* were found to have significantly elevated positive selection across all four homolog-level analyses (*i.e.*, with or without imposing a subpathway classification).

MK tests are commonly used to test for positive selection in individual genes. SnIPRE selection effects can be used to perform an analogous test for selection, except the approach can gain power by taking in the genome-wide distribution of polymorphism and divergence patterns by fitting gene as a random effect (Eilertson *et al.* 2010). We found that 36% of RNAi genes show nominally significant evidence for adaptive protein evolution across species analyzed. In contrast, only 5% of selection effects in control genes were significantly positive (Figure S6 in File S2). At the pathway level, 40% of piRNA genes, 44% of viRNA genes, 26% of nonantiviral siRNA-pathway genes, and 25% of miRNA-pathway genes had significantly positive selection effects (Figure S6 in File S2). No gene had positive selection effects in every lineage, although *armitage*, *capsuleen*, *cutoff*, *tudor*, *vasa*, *vretano*, and *Yb* homologs were identified in over half the lineages.

### Selective sweeps are detectable across functional classes of RNAi genes

**Figure 5:**
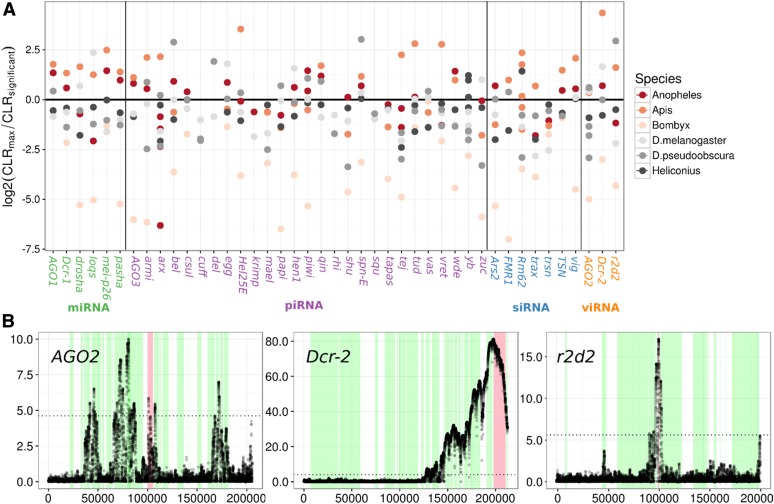
Selective sweeps in RNAi genes and example SweeD plots. (A) Points indicate the log_2_ ratio of the maximum observed CLR value (from SweeD) in the named gene to the CLR 95% significance threshold inferred from simulation. Values above zero indicate there was a significant CLR peak in a genic region and colors indicate species. (B) The viRNA pathway in *A. mellifera* shows strong evidence for recent sweeps. For each of the three viRNA-pathway genes, the CLR statistic is plotted across a 200-kb region. The dotted line is the significance threshold estimated through neutral simulations under a published demographic history. Red regions denote the focal gene and green regions highlight surrounding genes. In *Apis*, both *Dcr2* and *R2D2* show strong evidence for sweeps with the surrounding region of *Dcr2* being devoid of polymorphism, indicating this sweep was recent and rapid. *AGO2* also shows a significant peak, but this is narrow and only marginally significant.

Recent positive selection is expected to leave a characteristic mark in the genome, including a SFS skewed toward low and high frequency alleles and a local reduction in polymorphism (Smith and Haigh 1974; Barton 1998; Nielsen *et al.* 2005). As RNAi genes show elevated rates of adaptive evolution, we speculated that they may also exhibit more evidence of recent selective sweeps. Using SweeD, we found that many of the insect lineages do show evidence for sweeps in a subset of RNAi genes (Figure 5 and Figures S7–S14 in File S2). We tested whether RNAi genes have undergone more recent sweeps than surrounding genes by classifying nominally significant peaks as either occurring near (within 1 kb) an RNAi gene or not, and using a binomial test to determine whether more sweeps than expected occur in RNAi genes (given their length). In four of the six species tested (*D. melanogaster*, *D. pseudoobscura*, *A. mellifera*, and *A. gambiae*) there were significantly more detectable sweep signals in RNAi genes than in surrounding non-RNAi genes (*D. melanogaster*: *P* = 0.0006; *A. mellifera*: *P* = 0.015; *A. gambiae*: *P* = 0.0001; *D. pseudoobscura*: *P* = 7 × 10^−5^). However, we find no difference among subpathways in the frequency with which we detected recent sweeps. General differences in constraint between RNAi and control genes could bias these results, perhaps through misidentification of reduced diversity caused by elevated negative or background selection as a sweep. However, DFE-alpha and SnIPRE analyses suggest RNAi genes are less constrained (Figure 1 and Text S1 in File S1, Model 3A, nonsynonymous:piRNA effect), making our analysis conservative. None of the genes exhibited a significant CLR peak across all organisms tested, although *spn-E* and *vig* display significant evidence of recent sweeps in five of the six insect lineages. It was notable that 34% of the variation in the per-gene maximum CLR test statistic was attributable to species, consistent with either sample size or demographic history playing a substantial role in our power to detect sweeps.

Sweep signatures were the most pronounced in *A. mellifera*, in both the CLR magnitude and breadth of the genomic region affected (Figure 5 and Figure S12 in File S2). These were associated with large regions devoid of any polymorphism, despite the high rate of recombination seen in honeybees (Beye *et al.* 2006), which is expected to narrow the region affected by a nearby sweep. We also searched for evidence of haplotype structure, as would be expected during an ongoing or soft selective sweep using the nSL statistic (data not shown). However, there were no strong signals in any of the RNAi genes for which we had haplotype information.

## Discussion

Using both DFE-alpha and SnIPRE-like MK analyses, we identify elevated rates of adaptive evolution in RNAi-pathway genes across six insects and two nematodes. In most species, the RNAi-pathway genes were also more likely to display evidence of a recent selective sweep. As in *Drosophila*, genes involved in the suppression of viruses and TEs show the highest rates of adaptive evolution, consistent with these genes being engaged in an arms race in multiple invertebrate lineages. We were able to extend past *Drosophila* analyses by combining genic rates of adaptive evolution across species to infer positive selection associated with particular RNAi-pathway functions and homologs. We found accelerated adaptation across piRNA-pathway functions, including piRNA biogenesis machinery, effector proteins, and especially transcriptional silencing machinery. Although there was substantial variation in rates among RNAi genes, the antiviral genes *AGO2* and *Dcr-2* and the piRNA-pathway genes *maelstrom*, *eggless*, *piwi*, *aub*, *armitage*, *capsuleen*, *cutoff*, *tudor*, *vasa*, *vretano*, *spn-E*, *vig*, and *Yb* show consistently strong signatures of positive selection.

### Identification of rapidly evolving pathways by DFE-alpha and SnIPRE

Estimated rates of adaptive protein evolution in an MK-framework (McDonald and Kreitman 1991) can be biased by past population size changes and slightly deleterious mutations that segregate at low frequencies (McDonald and Kreitman 1991; Eyre-Walker 2002; Fay *et al.* 2002; Smith and Eyre-Walker 2002; Sawyer *et al.* 2003; Bierne and Eyre-Walker 2004; Welch 2006; Charlesworth and Eyre-Walker 2008; Eyre-Walker and Keightley 2009; Gossmann *et al.* 2012). Here we attempted to account for these biases by explicitly modeling the DFE and demographic history using DFE-alpha (Eyre-Walker and Keightley 2009), or by modeling the genome-wide patterns of polymorphism and divergence with SnIPRE (Eilertson *et al.* 2012). We expect these methods to complement one another. Both methods assume that demographic history affects all loci in a similar manner, but SnIPRE is better able to capture variability across loci in these effects, while DFE-alpha conditions on a point estimate. In addition, both methods can be biased by variation in the DFE, but under different circumstances. The DFE-alpha meta-analysis assumes a similar DFE for all genes, which may bias single-gene ω_A_ estimates in either direction, depending on the true DFE of a gene. SnIPRE, like traditional MK-style analyses, does not take into account the SFS, and so can be biased by slightly deleterious mutations which will downwardly bias selection effects. Therefore, for single-gene analyses SnIPRE is more conservative, while DFE-alpha can be more powerful if the SFS and DFE parameters are accurately estimated.

Most of the qualitative results of each of these analyses agree that genes in the piRNA and viRNA pathways are evolving adaptively. However, SnIPRE and DFE-alpha analyses disagree on the relative differences in the rate of adaptive evolution among subpathways. For example, the DFE-alpha meta-analysis provides low point estimates for the endo-siRNA and miRNA pathways relative to the piRNA and viRNA; but SnIPRE identifies the endo-siRNA selection effect as higher than the piRNA, and piRNA genes closer to the miRNA. As noted above, this incongruence could reflect differences in the DFE between subpathways; genes in the miRNA and endo-siRNA pathways are highly conserved and have low rates of protein evolution, while mechanisms of piRNA-pathway function are surprisingly diverse across animals (*e.g.*, Morazzani *et al.* 2012; Sarkies *et al.* 2015). These differences in constraint could lead to an underestimation of miRNA- and endo-siRNA-pathway adaptation and overestimation of piRNA adaptation in the DFE-alpha analyses, and could indicate that estimating the DFE separately for each subpathway may improve estimates.

### Adaptive protein evolution across species is enriched in specific functional pathways

We found large differences in rates of adaptive protein substitution between insects and nematodes, but less variation among insect species. In an ANOVA, we find that species explained only 11% of the variation in gene-level estimates of ω_A_, but gene and pathway explained 42% of the variation in gene-level ω_A_ estimates; suggesting that gene function is a greater determinant of the rate of adaptive evolution than species. The elevated rate seen in piRNA- and viRNA-pathway genes across species could be caused by rapid adaptation in the same subset of genes in a pathway, or in a random selection of genes in a pathway. Homolog-level analysis of ω_A_ and selection effects (Figure 4 and Figure S5 in File S2) indicates it is probably both because subsets of homologs within pathways show consistent evidence for elevated adaptive protein evolution, but homologous genes also exhibit high variances across species (but see Figure S4 in File S2).

Much of the variation in adaptive rate is not attributable to species or conserved gene function, and it is necessarily difficult to ascribe this remaining variance to a source. It is likely that the great majority is derived from the sampling error associated with measuring polymorphism and divergence in a single gene, however biological processes may also contribute. Functional divergence of a gene from its role in *Drosophila* could affect the adaptive rate in that species. For example, the repurposing of the piRNA pathway to target viruses in mosquitoes might be expected to increase adaptive rates of any factors shared in both anti-TE and antiviral roles (Morazzani *et al.* 2012). Additionally, if conflict is driving the observed adaptation, then differences in the magnitude or frequency of conflict could change the adaptive potential of a gene. In nature, this could be driven by differences in the diversity, frequency, or virulence of viral pathogens across species.

### Potential drivers of adaptation in the viRNA pathway

It seems likely that the elevated rates of adaptive protein evolution we detect in the viRNA and piRNA pathways are a result of recurrent selection mediated by viruses and/or TEs. First, it is well established that defensive pathways show high rates of adaptive evolution, presumably as a consequence of antagonistic coevolution with parasites (Stenseth and Smith 1984; Buckling and Rainey 2002; Paterson *et al.* 2010; Brockhurst *et al.* 2014). For example, a recent analysis of virus-interacting proteins estimated that 30% of adaptive protein changes in mammals are driven by viruses (Enard *et al.* 2016). Second, for the viRNA-pathway genes at least, viral suppressors of RNAi are strong candidates to be the driving agent. Many RNA and DNA viruses of invertebrates are known to have proteins or structural RNAs which actively block RNAi function (Li *et al.* 2002; Van Rij *et al.* 2006; Nayak *et al.* 2010; van Mierlo *et al.* 2012; Bronkhorst *et al.* 2014), and these can evolve rapidly and can be highly host specific, consistent with an arms-race scenario (van Mierlo *et al.* 2014). We find that *AGO2* and *Dcr-2* display consistently elevated rates of adaptive protein substitution across insect species, with additional limited evidence of elevated adaptation in *hen1*, all of which have previously been identified as targets of active suppression by viral proteins [viral suppressors of RNAi (VSRs)] (Van Rij *et al.* 2006; Vogler *et al.* 2007; Nayak *et al.* 2010; van Mierlo *et al.* 2012; van Cleef *et al.* 2014), lending credibility to the hypothesis that viruses may play a major role in driving the observed rapid evolution in RNAi genes.

### Potential drivers of adaptation in the piRNA pathway

Whereas an arms race between antiviral RNAi genes and viral suppressors of RNAi is intuitive, the observed rapid adaptive evolution of piRNA-pathway genes is currently harder to explain. Similar to viruses, TEs are costly for their hosts and could, in principle, select for increased suppression (Charlesworth *et al.* 1994). However, piRNA-generating clusters ostensibly provide an adaptive defense that can arise on much shorter timescales than fixation of advantageous mutations, reminiscent of acquired immunity (Brennecke *et al.* 2007; Khurana *et al.* 2011; Han *et al.* 2015; Mohn *et al.* 2015).

The adaptive response in piRNA genes could be mediated by at least three nonexclusive mechanisms: (i) direct piRNA-pathway suppression by TEs or by off-target VSRs, (ii) recurrent “retuning” of piRNA machinery after a novel TE invasion (Lee and Langley 2012; Yi *et al.* 2014), or (iii) fluctuating selection on the sensitivity to detect transposon sequences and specificity to exclude off-target genic silencing (*i.e.*, the “genomic auto-immune hypothesis”) (Blumenstiel *et al.* 2016). Besides the global derepression of transposons upon invasion of the Penelope retroelement in *D. virilis* (Petrov *et al.* 1995; Evgen’ev *et al.* 1997; Rozhkov *et al.* 2010; Blumenstiel *et al.* 2016), there is limited evidence for (i), and the mechanism underlying this phenomenon still awaits elucidation. The latter two hypotheses are not mutually exclusive, and both posit that piRNA adaptation occurs in response to recurrent horizontal transfer of new TEs into the genome, a common occurrence in insects (Peccoud *et al.* 2017). In (ii), the piRNA pathway evolves to optimize defense against the current suite of transposons, becoming “less adapted” for dealing with historic, obsolete ones. This would result in a Red Queen-like scenario, but instead of antagonistic coevolution with one parasite, the piRNA pathway must defend against a constant recycling of TE lineages. As the germline cells face a higher TE diversity than somatic tissues, this is broadly supported by our observation that piRNA-pathway genes with primarily germline function (Czech *et al.* 2013; Handler *et al.* 2013; Muerdter *et al.* 2013) have higher rates of adaptive protein evolution than those functioning in the somatic layer of cells surrounding the *Drosophila* ovary (Figure S15 in File S2); although this is difficult to disentangle from previous observations that germline-specific genes have generally high adaptive rates (Choi and Aquadro 2015; Flores *et al.* 2015). The genomic autoimmunity hypothesis (iii) goes further and proposes piRNA-pathway adaptation to TE invasions results in increased piRNA function and associated off-target genic effects, which are then selected against after the TE is suppressed (Castillo *et al.* 2011; Blumenstiel *et al.* 2016). It could be argued that our analysis of adaptive rates in piRNA functions lends broad support for this, in that genes mediating transcriptional silencing show the greatest adaptive rates across species in the piRNA pathway, with additional evidence for rapid adaptation in biogenesis factors whose rates are expected to be correlated with the transcriptional machinery (Blumenstiel *et al.* 2016). However, our pathway-level and homolog-level analyses also find signals of elevated adaptation in effector genes, which have rates that covary to a lesser degree with other piRNA factors (Blumenstiel *et al.* 2016). This does not refute the genomic autoimmunity hypothesis, but may suggest additional selective forces acting on the piRNA pathway, independent of genes, underlying a trade-off between sensitivity and specificity. Nevertheless, our results would also fit within the context of (ii) in a scenario where the transcriptional machinery has a greater evolutionary potential than the rest of the piRNA pathway.

### Concluding remarks

Accelerated adaptive evolution in RNAi genes has been described in multiple *Drosophila* species, where a subset of genes evolve adaptively in siRNA and piRNA pathways, but not the miRNA pathway. Our analyses extend the observation of rapid RNAi gene evolution, and generalize elevated positive selection in piRNA and viRNA pathways across six insect and two nematode species.

## Supplementary Material

Supplemental material is available online at www.genetics.org/lookup/suppl/doi:10.1534/genetics.117.300567/-/DC1.
