# Supplementary material for: RNA-Interference Pathways Display High Rates of Adaptive Protein Evolution in Multiple Invertebrates
Source: Genetics. 2018 Feb 1;208(4):1585–99. doi: 10.1534/genetics.117.300567 (PMC5887150; doi:10.1534/genetics.117.300567)
Supplement: Supplementary file 5 [file 1585FileS1.docx]

S1 Text: Supplementary R code for models

*DFE-alpha analyses*

DFE-alpha (Eyre-Walker & Keightley, 2009) infers ω_A_ (the number of adaptive nonsynonymous substitutions per nonsynonymous site, relative to the number of synonymous substitutions per synonymous site), while simultaneously modelling the distribution of deleterious fitness effects and a population size change (Keightley & Eyre-Walker, 2007; Eyre-Walker & Keightley, 2009). The ω_A_ statistic is closely related to the more widely reported α statistic (Charlesworth, 1994; Fay, et al., 2001; Fay et al, 2002; Smith & Eyre-Walker, 2002; Bierne & Eyre-Walker, 2004; Welch, 2006), but differs in that ω_A_ is expected to be less dependent on effective population size and therefore better for cross-species comparisons. This is because the denominator, dS, should be less affected by the efficacy of selection, and thus effective population size (Gossmann, et al., 2010; Gossmann, et al., 2012; Kousathanas, et al., 2014). DFE-alpha utilises the observed site frequency spectrum (SFS) for putatively unconstrained synonymous sites and potentially selected nonsynonymous sites, and maximises the likelihood of observing these spectra given the distribution of deleterious fitness effects (DFE) for nonsynonymous variants and a step-change in effective population size (Eyre-Walker & Keightley, 2009). The ‘excess’ nonsynonymous divergence attributable to adaptive substitution is then inferred, given the maximum likelihood estimate of the DFE and the observed divergence (Eyre-Walker & Keightley, 2009). We inferred ω_A_ for: (i) each RNAi gene and each position-matched ‘control’ gene (i.e. those with no known RNAi-pathway role falling within the same 200 Kbp interval); (ii) each RNAi subpathway and their respective control genes, and; (iii) all RNAi pathway genes together, by pooling polymorphism and divergence data across genes within classes. We then compared this grouped polymorphism and divergence data in pathways of interest against control genes. We estimated the parameters of the nominal change in population size (the relative population size change parameter N_2_, and the time of the population size change, t_2_) for all genes treated together within species, and then fixed these estimates for pathway and individual gene estimates. Conditional on this species-wide estimate of demographic history, the DFE was estimated separately for RNAi and control genes. We obtained confidence intervals for estimates of α and ω_A_ by bootstrapping genes within classes (1000 draws), and we tested for differences in rate between gene classes by randomly permuting genes 1000 times between classes. To test for differences in the DFE between RNAi and control genes we performed a likelihood ratio test between a null model in which parameters of the DFE were estimated for all genes together, and an alternative one in which we allowed the DFE parameters to be estimated separately for RNAi and control genes, holding all other parameters constant.

Pooling polymorphism and divergence data across genes allows calculation of pathway-specific ω_A_ within a species, but cannot readily give cross-species estimates. Further, underlying unaccounted-for structure in the data could bias results, for example, if a high divergence gene has few polymorphisms, then it will influence divergence, but not DFE, estimation. Therefore, we also calculated ω_A_ for individual genes in each species, and analysed these estimates across species. In general, such estimates are extremely poor unless samples sizes are extremely large (e.g. hundreds of alleles are sampled, or genes are very large) (Keightley & Eyre-Walker, 2010). However, if the selective pressure acting on genes is consistent across species, as assumed by many phylogenetic approaches (Yang, 2007), we can acquire more accurate estimates of the relative rate of adaptive evolution by combining information across species. We therefore used a formal meta-analytic approach to combine small-group and single-gene estimates across species by constructing linear mixed models using MCMCglmm (Hadfield, 2010). These models were used to estimate average gene-level ω_A_ of pathways and homologues, and variation among gene-level ω_A_ estimates.

The first three models took the same form, only distinguished by the pathways among which genes were divided. In Model 1A the genes were classified as either ‘control’ or ‘RNAi’, in Model 1B the RNAi class was expanded into four levels: ‘miRNA’, ‘siRNA’, ‘viRNA’, and ‘piRNA’ and in Model 1C the piRNA class was further split into three functional categories: ‘effectors of transcriptional silencing’, ‘effectors of post-transcriptional silencing’, and ‘biogenesis factors’. The model for the estimate of $\omega_{A}$ (i.e. $\hat{\omega}_{A}$) for homologue *k* in gene class *l* in species *m* had the form:

$\hat{\omega}_{A:klm}=\beta_{0}+\beta_{Class:l}+u_{Organism:m}+m_{klm}+\varepsilon_{klm}$ [1]

where $\beta_{0}$ is the intercept, $\beta_{Class:l}$ is a fixed effect associated with gene class *l*, $u_{Organism:m}$ is a random effect associated with species *m*, $m_{klm}$ is the sampling error associated with each estimate, and $\varepsilon_{klm}$ is the between observation error after accounting for measurement error, which was allowed to vary by gene class (i.e. pathway). The variance of the sampling errors was obtained by bootstrapping genes by codon, and this sampling error variance was fixed at that value in the analysis. All species effects were assumed to come from a single normal distribution but the errors were assumed to come from independent normal distributions with different variances for each gene class.

Model 2 extended Model 1 by including homologue as a random effect ($u_{Hom:kl}$) in order to identify homologues with elevated adaptation across lineages, where each homologue *k* is represented by approximately 6 genes, one from each insect species analysed, except for the cases where an orthologue was not present in every species (resulting in fewer than 6 observations for a homologue), or a gene had duplicated in one or more of the species (resulting in greater than 6 observations for a homologue).

$\hat{\omega}_{A:klm}=\beta_{0}+\beta_{Class:l}+u_{Organism:m}+{u_{Hom:kl}+ m}_{klm}+\varepsilon_{klm}$ [2]

Here, the homologue effects were assumed to come from independent normal distributions with different variances for each gene class. In this model the cross-species average $\omega_{A}$ for a homologue *k* in gene class *l* is given by $\bar{\omega}_{A:kl}= {\beta_{0}+u}_{Class:l}+u_{Hom:kl}$. However, if genes are misclassified with respect to the gene class they belong, then $\bar{\omega}_{A:kl}$ is likely to biased in general, and particularly so for misclassified genes. An arguably more conservative approach is to only use information from homologous genes to estimate the cross-species (i.e. remove the class effects from the model; this approach is provided as Model 2B in S1 text) and have $\bar{\omega}_{A:kl}= {\beta_{0}+u}_{Hom:kl}$. See below for R code and a full description of the models used.

*SnIPRE-like analysis*

The meta-analytic approach to cross-species analysis above has the advantage of utilising DFE-alpha estimates that are inferred under an explicit population-genetic model. However, it has the disadvantage that it conditions on point estimates from a model, rather than using the available data directly. We have therefore taken advantage of the Poisson linear mixed model approach to MK analyses ‘SnIPRE’ proposed by Eilertson et al. (2012), which models the counts of mutations in four classes: synonymous within-species polymorphisms, nonsynonymous within-species polymorphisms, between-species synonymous differences (divergence) and between-species nonsynonymous differences. By ﬁtting ‘nonsynonymous’ and ‘divergent’ as main effects, selection can be inferred from their interaction, which records the excess contribution of nonsynonymous mutations to between-species divergence. This excess can be assessed at the level of individual genes (by treating gene identity as a random effect) or can be expressed as a function of other ﬁxed or random effects such as gene class and species. Although this approach does not directly provide parameter estimates that are interpretable in simple population-genetic terms, such as ω_A_, it has the advantage of extending naturally to provide comparisons between species and gene classes while still using raw count data directly. Here we combine polymorphism and divergence data from several species to test whether RNAi genes have higher rates of adaptive substitution than our set of control genes, whether these rates vary between different subclasses of RNAi gene, and whether these rates vary between different homologues. We fitted these models with the R package MCMCglmm (Hadfield, 2010) and the code is provided in the S1 text. In their single-species and single-class analysis Eilertson et al. (2012) used the generalised linear mixed model with the ﬁxed effect part of the model as:

$log\left( \mu_{ijk} \right)=\beta_{0}+\beta^{N}i+\beta^{D}j+\beta^{ND}ij{+ \beta}_{length}x_{ik}$ [3]

where $\mu_{ik}$ is the expected number of mutations in gene *k* in one of the four classes indexed by *i* = 0,1 and *j* = 0,1 where *i* = 1 indicates nonsynonymous (*N*) and *k* = 1 divergent (*D*). This model estimates the intercept $\beta_{0}$ (the density of synonymous polymorphisms), $\beta^{N}$ (the genome-wide difference between a mutation being nonsynonymous versus synonymous),$\beta^{D}$ (the genome-wide difference between a mutation being a substitution versus a polymorphism), and $\beta^{ND}$ (the interaction effect describing any genome-wide excess or dearth of nonsynonymous substitutions). Parameter $x_{ik}$ is the logarithm of the number of sites in gene *k* where a synonymous (*i* = 0) or a nonsynonymous (*i* = 1) mutation could occur and the fixed effect$\beta_{length}$ models how the number of observed mutations changes as a function of the number of sites. Eilertson et al. (2012) also fitted a random effect structure that models between-gene mutation patterns after accounting for the ﬁxed effects:

$log\left( \mu_{ijk} \right)=\beta_{0}+\beta^{N}i+\beta^{D}j+\beta^{ND}ij+\beta_{length}x_{ik}+\varepsilon_{k}+ \varepsilon_{k}^{N}i+\varepsilon_{k}^{D}j+\varepsilon_{k}^{ND}ij$ [4]

where the additional terms denoted $\varepsilon$ are the gene-specific random deviations from each of the first four fixed effect terms described above. The four gene-specific random deviations were assumed to come from a multivariate normal distribution with estimated (co)variance matrix. Eilertson et al. (2012) define the selection effect of gene *k* as ${\beta^{ND}+\varepsilon}_{k}^{DG}$ , where a positive effect is evidence for positive selection, and (in Bayesian terms) the posterior probability that the effect exceeds zero can be directly assessed.

Here we extend the *SnIPRE*-like model of Eilertson et al. (2012) to accommodate multiple species and to allow the evolutionary parameters to differ among different classes of gene. To this end we allowed the four fixed effects to vary by species and by gene class (control, piRNA, siRNA, miRNA and viRNA) to give the fixed effect model:

${\beta_{0}+\beta}^{N}i+\beta^{D}j+\beta^{ND}ij+{\beta_{length}x_{iklm}+\beta}_{Class:l}+ \beta_{Class:l}^{N}i+\beta_{Class:l}^{D}j+\beta_{Class:l}^{ND}ij+ \beta_{Organism:m}+ \beta_{Organism:m}^{N}i+\beta_{Organism:m}^{D}j+\beta_{Organism:m}^{ND}ij$ [5]

From this we calculated the estimated selection effect for a specific pathway as $\beta^{ND}+\beta_{Class:l}^{ND}$. The random effect portion of the model included homologue-specific effects and gene-specific effects and had the form

$u_{Hom:k}+ u_{Hom:k}^{N}i+u_{Hom:k}^{D}j+u_{Hom:k}^{ND}ij+ \varepsilon_{klm}+ \varepsilon_{klm}^{N}i+\varepsilon_{klm}^{D}j+\varepsilon_{klm}^{ND}ij$ [6]

In addition to the four gene effects, the four homologue effects were also assumed to come from a multivariate normal distribution with estimated (co)variance matrix. We used this model to calculate the selection effect for homologue *k* in gene class *l* as $\beta^{ND}+\beta_{Class:l}^{ND}+$ $u_{Hom:kl}^{ND}$and each gene as $\beta^{ND}+\beta_{Class:l}^{ND}+$ $u_{Hom:klm}^{ND}$+ $\varepsilon_{klm}^{ND}$. We estimated $\beta_{length}$ rather than fixing it at one, as in Eilertson et al. (2012), although the posterior mean of $\beta_{length}$ was close to one, supporting the assumption of Eilertson et al. (2012). In addition, we also fitted the SnIPRE model without assuming genes belong to known pathways, analogous to model 2. The code to fit these models is provided in the S1 text.

DFE-alpha meta-analysis

Data set up:

library(MCMCglmm)

## Loading required package: Matrix

## Loading required package: coda

## Loading required package: ape

#########################################################
# Data Upload for gene-level omega.A linear mixed models -------------------------------------------------------------
dat<-read.table("dfe-alpha-ind_withVIRNA.csv", sep=",", header=TRUE)
dat$omega_A[which(dat$omega_A==-Inf)]<-NA #Remove genes that can't be estimated. The results are unaffected if these values are set to zero.
dat<-subset(dat, !is.na(omega_A) & !is.na(omega_se) )

Model 1A: Comparison of RNAi and control genes

prior.1A=list(R=list(V=diag(2),
 nu=0.002),
 G=list(G1=list(V=diag(1),
 nu=1,
 alpha.mu=c(0),
 alpha.V=diag(1))))

model.1A <-MCMCglmm(omega_A~Class,
 random= ~Organism,
 rcov = ~idh(Class):units,
 mev=dat$omega_se^2,
 prior=prior.1A,
 data=dat, verbose = FALSE)

Model 1A models gene-level ωA estimates as a gaussian response with gene class as a fixed effect and species as a random effect. For the random effects (random= ~Organism), we assume all (co)variances among organisms are equal. We also estimate separate error variances for each gene class (rcov = ~idh(Class):units), allowing us to test whether the variance of the adaptive rate of RNAi and control genes differ. The idh() function specifies that the residual variance associated with each class of genes is independent, and sets the off-diagonals of the covariance matrix to zero. We specify the sampling error associated with each estimate of ωA (mev=dat$omega_se^2) obtained by bootstrapping by codon and rerunning DFE-alpha on the new codon set.

summary(model.1A)

##
## Iterations = 3001:12991
## Thinning interval = 10
## Sample size = 1000
##
## DIC: -1537.862
##
## G-structure: ~Organism
##
## post.mean l-95% CI u-95% CI eff.samp
## Organism 8.451e-05 1.994e-10 0.0002989 518.6
##
## R-structure: ~idh(Class):units
##
## post.mean l-95% CI u-95% CI eff.samp
## ClassControl.units 0.0003228 0.0001796 0.0004817 705.2
## ClassRNAi.units 0.0036677 0.0021694 0.0052551 596.8
##
## Location effects: omega_A ~ Class
##
## post.mean l-95% CI u-95% CI eff.samp pMCMC
## (Intercept) 0.0095622 0.0002037 0.0188204 1000 0.028 *
## ClassRNAi 0.0530510 0.0382375 0.0662674 1000 <0.001 ***
## ---
## Signif. codes: 0 '***' 0.001 '**' 0.01 '*' 0.05 '.' 0.1 ' ' 1

The command summary(model.1A) prints some aspects of the MCMC chain, the DIC score, the variance components for the G-structure (random effects), the error variance estimates, and the estimates for the fixed effects. The RNAi class of genes is estimated to be 0.05 greater than control genes, and this is signficant (pMCMC < 0.001) We also test whether the variance is significantly greater for RNAi genes using the posterior distributions for the error variances saved in the VCV object.

head(model.1A$VCV)[,c("ClassControl.units", "ClassRNAi.units")]

## Markov Chain Monte Carlo (MCMC) output:
## Start = 3001
## End = 3061
## Thinning interval = 10
## ClassControl.units ClassRNAi.units
## [1,] 0.0002867517 0.002768112
## [2,] 0.0002296532 0.004182672
## [3,] 0.0002213186 0.002911138
## [4,] 0.0002727306 0.003119912
## [5,] 0.0002874699 0.002956077
## [6,] 0.0003067956 0.004262320
## [7,] 0.0002863971 0.002101608

To test for significantly different variances, we subtract one posterior distribution from the other and ask what proportion overlaps zero.

iterations.less.than.zero <- length(which(model.1A$VCV[,"ClassControl.units"] - model.1A$VCV[,"ClassRNAi.units"] > 0))
total.chain.length <- nrow(model.1A$VCV)
pMCMC <- iterations.less.than.zero/total.chain.length
pMCMC*2

## [1] 0

Therefore, in every iteration of the chain (1000 sampled iterations, thinning interval of 10), the error variance associated with RNAi genes was greater than control genes (MCMCp < 0.001).

Model 1B: Comparison of RNAi subpathways (piRNA, siRNA, viRNA, miRNA)

prior.1B=list(R=list(V=diag(5),
 nu=0.002),
 G=list(G1=list(V=diag(1),
 nu=1,
 alpha.mu=c(0),
 alpha.V=diag(1))))
model.1B <-MCMCglmm(omega_A~Subclass,
 random= ~Organism,
 data=dat,
 mev=dat$omega_se^2,
 rcov = ~idh(Subclass):units,
 prior=prior.1B, verbose = FALSE)

Model 1B is similar to model 1A, except the RNAi class has now been divided into four subpathways (miRNA, siRNA, piRNA, viRNA). The summary of the model output is the following:

summary(model.1B)

##
## Iterations = 3001:12991
## Thinning interval = 10
## Sample size = 1000
##
## DIC: -1529.198
##
## G-structure: ~Organism
##
## post.mean l-95% CI u-95% CI eff.samp
## Organism 7.741e-05 4.421e-11 0.0003035 673.8
##
## R-structure: ~idh(Subclass):units
##
## post.mean l-95% CI u-95% CI eff.samp
## SubclassControl.units 0.0003252 0.0001822 0.0004842 553.1
## Subclassmi.units 0.0006925 0.0002427 0.0012781 990.4
## Subclasspi.units 0.0063899 0.0037257 0.0094438 661.8
## Subclasssi.units 0.0012648 0.0003426 0.0024599 1000.0
## Subclassvi.units 0.0391034 0.0081908 0.0842066 1000.0
##
## Location effects: omega_A ~ Subclass
##
## post.mean l-95% CI u-95% CI eff.samp pMCMC
## (Intercept) 0.0096087 0.0002142 0.0178675 902.3 0.036 *
## Subclassmi 0.0108465 -0.0007559 0.0246557 1000.0 0.082 .
## Subclasspi 0.0794998 0.0586724 0.1002338 1000.0 <0.001 ***
## Subclasssi 0.0301952 0.0087547 0.0480110 1000.0 0.002 **
## Subclassvi 0.1863027 0.0690058 0.3184570 1000.0 0.002 **
## ---
## Signif. codes: 0 '***' 0.001 '**' 0.01 '*' 0.05 '.' 0.1 ' ' 1

The subclass effects (parameterised as "mi", "pi", "si", and "vi"), along with their pMCMC values are listed under location effects. Differences in residual variances between subpathways can be tested in a similar manner to model 1A. We tested whether certain subpathways were greater than others as previously done with variance components in Model 1A, except using the posteriors for the fixed effects stored in the Sol object. For example:

iterations.less.than.zero <- length(which(model.1B$Sol[,"Subclassmi"] - model.1B$Sol[,"Subclassvi"] > 0))
total.chain.length <- nrow(model.1B$Sol)
pMCMC <- iterations.less.than.zero/total.chain.length
pMCMC*2

## [1] 0.002

We conclude that the viRNA pathway has a significantly greater rate of adaptive protein evolution (MCMCp = 0.002).

Model 1C: Comparison of RNAi subpathways, with the piRNA split into effectors, biogenesis factors, and transcriptional silencing factors.

dat$Subclass_pi <- factor(dat$Subclass_pi, levels=c("Control", "mi", "si","effector", "transcriptional", "biogenesis","vi"))

prior.1C=list(R=list(V=diag(7), nu=0.002),
 G=list(G1=list(V=diag(1), nu=1, alpha.mu=c(0), alpha.V=diag(1))))
model.1C <-MCMCglmm(omega_A~Subclass_pi,
 random= ~Organism,
 rcov = ~idh(Subclass_pi):units,
 mev=dat$omega_se^2,
 data=dat,
 prior=prior.1C, verbose = FALSE, nitt = 50000)

Again, Model 1C is structurally identical to Model 1A and Model 1B, with the only difference being the number of factor levels which genes are grouped into.

summary(model.1C)

##
## Iterations = 3001:49991
## Thinning interval = 10
## Sample size = 4700
##
## DIC: -1533.084
##
## G-structure: ~Organism
##
## post.mean l-95% CI u-95% CI eff.samp
## Organism 6.068e-05 1.341e-11 0.0002115 2955
##
## R-structure: ~idh(Subclass_pi):units
##
## post.mean l-95% CI u-95% CI eff.samp
## Subclass_piControl.units 0.0003235 0.0001818 0.0004742 3475
## Subclass_pimi.units 0.0005880 0.0002091 0.0010588 4254
## Subclass_pisi.units 0.0012564 0.0003701 0.0024096 4342
## Subclass_pieffector.units 0.0071395 0.0017279 0.0144296 4700
## Subclass_pitranscriptional.units 0.0158533 0.0003308 0.0397985 2898
## Subclass_pibiogenesis.units 0.0069787 0.0032832 0.0112018 3689
## Subclass_pivi.units 0.0391355 0.0080485 0.0820324 4700
##
## Location effects: omega_A ~ Subclass_pi
##
## post.mean l-95% CI u-95% CI eff.samp pMCMC
## (Intercept) 0.009687 0.001916 0.018081 4132 0.02383
## Subclass_pimi 0.010512 -0.001300 0.022172 4700 0.06638
## Subclass_pisi 0.030278 0.011611 0.051474 4315 0.00383
## Subclass_pieffector 0.079908 0.036203 0.125608 4700 < 2e-04
## Subclass_pitranscriptional 0.154907 0.074015 0.242532 4013 < 2e-04
## Subclass_pibiogenesis 0.078657 0.054069 0.106494 4700 < 2e-04
## Subclass_pivi 0.189578 0.067866 0.309402 5537 0.00340
##
## (Intercept) *
## Subclass_pimi .
## Subclass_pisi **
## Subclass_pieffector ***
## Subclass_pitranscriptional ***
## Subclass_pibiogenesis ***
## Subclass_pivi **
## ---
## Signif. codes: 0 '***' 0.001 '**' 0.01 '*' 0.05 '.' 0.1 ' ' 1

All three piRNA pathways are significantly greater than control genes. Significance between subpathways and variance components was assessed as above.

Model 2A: Comparison of RNAi homologues (with subpathway as a fixed effect)

prior.2A=list(R=list(V=diag(5), nu=0.002),
 G=list(G1=list(V=diag(4),
 nu=1,
 alpha.mu=rep(0,4),
 alpha.V=diag(4)),
 G2=list(V=diag(1),
 nu=1,
 alpha.mu=rep(0,1),
 alpha.V=diag(1))))
model.2A <-MCMCglmm(omega_A~Subclass,
 random=~idh(at.level(Subclass, c('mi', 'pi', 'si', 'vi'))):Gene + Organism,
 rcov =~idh(Subclass):units,
 data=dat, mev=dat$omega_se^2, pr=TRUE, prior = prior.2A, verbose = FALSE)

The second model is similar to the first, but for two differences. First, the effect of each RNAi homologue is estimated across species, specifying idh(at.level(Subclass, c('mi', 'pi', 'si', '.vi'))) so that the homologue effect is not estimated for the (nonhomologous) control genes. Second, we specify pr=TRUE, so that the random effects are stored along with the fixed effects in the model output.

summary(model.2A)

##
## Iterations = 3001:12991
## Thinning interval = 10
## Sample size = 1000
##
## DIC: -1542.535
##
## G-structure: ~idh(at.level(Subclass, c("mi", "pi", "si", "vi"))):Gene
##
## post.mean l-95% CI
## at.level(Subclass, c("mi", "pi", "si", "vi"))1.Gene 0.0004272 1.394e-11
## at.level(Subclass, c("mi", "pi", "si", "vi"))2.Gene 0.0006833 8.664e-09
## at.level(Subclass, c("mi", "pi", "si", "vi"))3.Gene 0.0005370 2.340e-13
## at.level(Subclass, c("mi", "pi", "si", "vi"))4.Gene 0.1036248 2.972e-08
## u-95% CI eff.samp
## at.level(Subclass, c("mi", "pi", "si", "vi"))1.Gene 0.001419 649.3
## at.level(Subclass, c("mi", "pi", "si", "vi"))2.Gene 0.002115 1000.0
## at.level(Subclass, c("mi", "pi", "si", "vi"))3.Gene 0.002051 438.2
## at.level(Subclass, c("mi", "pi", "si", "vi"))4.Gene 0.325240 1000.0
##
## ~Organism
##
## post.mean l-95% CI u-95% CI eff.samp
## Organism 6.993e-05 9.763e-11 0.0002299 514.5
##
## R-structure: ~idh(Subclass):units
##
## post.mean l-95% CI u-95% CI eff.samp
## SubclassControl.units 0.0003251 0.0001810 0.0004723 698.9
## Subclassmi.units 0.0005521 0.0001729 0.0010869 771.4
## Subclasspi.units 0.0060251 0.0033111 0.0094265 688.9
## Subclasssi.units 0.0012674 0.0003819 0.0025295 1000.0
## Subclassvi.units 0.0431069 0.0083074 0.0930210 1000.0
##
## Location effects: omega_A ~ Subclass
##
## post.mean l-95% CI u-95% CI eff.samp pMCMC
## (Intercept) 0.009849 0.002151 0.018498 1000 0.024 *
## Subclassmi 0.011261 -0.006067 0.033797 1000 0.220
## Subclasspi 0.081436 0.058334 0.103126 1000 <0.001 ***
## Subclasssi 0.031000 0.005495 0.065159 1000 0.044 *
## Subclassvi 0.188296 -0.077555 0.471909 1000 0.108
## ---
## Signif. codes: 0 '***' 0.001 '**' 0.01 '*' 0.05 '.' 0.1 ' ' 1

The posterior distribution of the fixed and random effects of the model are stored in the object named model.2A$Sol, with columns pertaining to each distribution. For example:

colnames(data.frame(model.2A$Sol))[c(1:10, 44:50)] #Show columns of interest

## [1] "X.Intercept."
## [2] "Subclassmi"
## [3] "Subclasspi"
## [4] "Subclasssi"
## [5] "Subclassvi"
## [6] "at.level.Subclass..c..mi....pi....si....vi...1.Gene.ago1"
## [7] "at.level.Subclass..c..mi....pi....si....vi...1.Gene.ars2"
## [8] "at.level.Subclass..c..mi....pi....si....vi...1.Gene.dcr1"
## [9] "at.level.Subclass..c..mi....pi....si....vi...1.Gene.drosha"
## [10] "at.level.Subclass..c..mi....pi....si....vi...1.Gene.loqs"
## [11] "at.level.Subclass..c..mi....pi....si....vi...3.Gene.tsn"
## [12] "at.level.Subclass..c..mi....pi....si....vi...3.Gene.vig"
## [13] "at.level.Subclass..c..mi....pi....si....vi...4.Gene.ago2"
## [14] "at.level.Subclass..c..mi....pi....si....vi...4.Gene.dcr2"
## [15] "at.level.Subclass..c..mi....pi....si....vi...4.Gene.r2d2"
## [16] "Organism.Anopheles"
## [17] "Organism.Apis"

We obtain the posterior distribution for ωA of an individual homologue by adding the posterior distributions for the intercept, subclass, and homologue. For example, the ωA posterior for Argonaute-2 is:

ago2.posterior <- model.2A$Sol[,"(Intercept)"] + model.2A$Sol[,"Subclassvi"] + model.2A$Sol[,"at.level(Subclass, c(\"mi\", \"pi\", \"si\", \"vi\"))4.Gene.ago2"]

We obtain 95% HPD confidence intervals using the command HPDinterval():

HPDinterval(ago2.posterior)

## lower upper
## var1 0.05833242 0.4022962
## attr(,"Probability")
## [1] 0.95

This show the lower 95% HPD interval (0.07) is greater than 0. We test whether this is greater than control genes by subtracting the posterior of ωA estimates of Argonaute-2 from the control gene class posterior, and see the proportion of MCMC intervals where it overlaps zero.

control.posterior <- model.2A$Sol[,"(Intercept)"]
control.minus.ago2.posterior <- control.posterior - ago2.posterior

iterations.less.than.zero <- length(which(control.minus.ago2.posterior > 0))
total.chain.length <- length(control.minus.ago2.posterior)
pMCMC <- iterations.less.than.zero/total.chain.length
pMCMC*2 #Multiply by 2 to make a two tailed test

## [1] 0.012

We conclude Argonaute-2 has a greater adaptive rate than control genes (pMCMC = 0.012).

Model 2B: Comparison of RNAi genes

prior.2B=list(R=list(V=diag(1), nu=0.002),
 G=list(G1=list(V=diag(1),
 nu=1,
 alpha.mu=rep(0,1),
 alpha.V=diag(1)),
 G2=list(V=diag(1),
 nu=1,
 alpha.mu=rep(0,1),
 alpha.V=diag(1))))
model.2B <-MCMCglmm(omega_A~1,
 random=~idv(at.level(Class, c('RNAi'))):Gene + Organism,
 data=dat, mev=dat$omega_se^2, pr=TRUE, prior = prior.2B, verbose=FALSE, nitt = 50000)

We also parameterise the second model without assigning genes to subpathways, and remove the subclass fixed effect and subclass-specific error-variances. The following is the output of the model:

summary(model.2B)

##
## Iterations = 3001:49991
## Thinning interval = 10
## Sample size = 4700
##
## DIC: -1749.396
##
## G-structure: ~idv(at.level(Class, c("RNAi"))):Gene
##
## post.mean l-95% CI u-95% CI eff.samp
## at.level(Class,c("RNAi")).Gene 0.003086 0.001022 0.005389 973
##
## ~Organism
##
## post.mean l-95% CI u-95% CI eff.samp
## Organism 0.0003451 5.648e-06 0.00105 917.2
##
## R-structure: ~units
##
## post.mean l-95% CI u-95% CI eff.samp
## units 0.0006735 0.0003879 0.0009833 1893
##
## Location effects: omega_A ~ 1
##
## post.mean l-95% CI u-95% CI eff.samp pMCMC
## (Intercept) 0.019337 0.002746 0.035655 4089 0.0281 *
## ---
## Signif. codes: 0 '***' 0.001 '**' 0.01 '*' 0.05 '.' 0.1 ' ' 1

Without a subclass effect, we assess whether a gene has a significantly elevated rate of adaptive amino acid evolution by comparing the posterior distributions of each gene effect to zero. For example, for Argonaute-2:

ago2.posterior <- model.2B$Sol[,"at.level(Class, c(\"RNAi\")).Gene.ago2"]
pMCMC <- length(which(ago2.posterior < 0))/length(ago2.posterior)
pMCMC*2

## [1] 0.04723404

We conclude that Argonaute-2 has significantly elevated ωA (pMCMC = 0.047).

SnIPRE-like analysis

Model 3A: SnIPRE-like analysis, with subpathway as a fixed effect

Set up data

library(MCMCglmm)
library(MASS)
dat<-read.csv("C:/Users/willi/Desktop/xspecies_rnaigene_counts_withpiRNA.csv", sep = " ")
dat$nfac<-as.factor(paste(dat$divergence, dat$nonsynonymous))
dat$length<-dat$Ln
dat$length[which(dat$synonymous==1)]<-dat$Ls[which(dat$synonymous==1)]
dat$gene.id<-paste(dat$organism, dat$gene, dat$Duplicate)
dat<-dat[-which(dat$organism=="bombyx" & dat$gene=="vas"),] #This gene had a problematic alignment
missing<-table(dat$gene.id,dat$nfac)
missing<-missing[which(rowSums(missing)!=4),] #Remove genes with missing data

for(i in 1:nrow(missing)){
 combi<-strsplit(rownames(missing)[i], " ")[[1]]
 combj<-which(missing[i,]==0)

 for(j in 1:length(combj)){
 dat[nrow(dat)+1,]<-dat[which(dat$organism==combi[1] & dat$gene==combi[2] & dat$Duplicate==combi[3])[1],]
 dat[nrow(dat),"nfac"]<-colnames(missing)[combj[j]]
 dat[nrow(dat),"count"]<-0
 dat[nrow(dat),"length"]<-1
 dat[nrow(dat),"divergence"]<-as.numeric(substr(colnames(missing)[combj[j]],1,1))
 dat[nrow(dat),"nonsynonymous"]<-as.numeric(substr(colnames(missing)[combj[j]],3,3))
 }
}
rownames(dat)<-1:nrow(dat)
dat$organism <- factor(dat$organism, levels = c("dmel", "anopheles", "apis", "bombyx", "dpse", "heliconius"))

The data look like the following. Each gene in each species is represented by 4 rows of count data, one for each of the MK observations of polymorphism and divergence by synonymous and nonsynonymous mutation types.

head(dat[dat$RNAi==1,c(1,2,3,6,7,10,12:19)])

## gene count divergence nonsynonymous organism Duplicate piRNA siRNA
## 465 ago1 3 1 1 anopheles A 0 0
## 466 ago1 10 1 0 anopheles A 0 0
## 467 ago1 8 0 0 anopheles A 0 0
## 468 ago1 2 0 1 anopheles A 0 0
## 469 ago1 0 1 1 apis A 0 0
## 470 ago1 39 1 0 apis A 0 0
## miRNA viRNA effector transcriptional biogenesis nfac
## 465 1 0 0 0 0 1 1
## 466 1 0 0 0 0 1 0
## 467 1 0 0 0 0 0 0
## 468 1 0 0 0 0 0 1
## 469 1 0 0 0 0 1 1
## 470 1 0 0 0 0 1 0

prior.3A<-list(R=list(V=diag(4), nu=0.002),
 G=list(G1=list(V=diag(4),
 nu=4,
 alpha.mu=rep(0,4),
 alpha.V=diag(4)*1000)))
model.3A<-MCMCglmm(count~log(length) + nonsynonymous + divergence + nonsynonymous:divergence + organism +
 (nonsynonymous + divergence + nonsynonymous:divergence):(organism + piRNA + viRNA + miRNA + siRNA),
 random=~us(1 + nonsynonymous + divergence + nonsynonymous:divergence):gene,
 rcov=~us(nfac):organism:gene:Duplicate, family="poisson",
 data=dat, pr = TRUE, pl=TRUE, prior = prior.3A, verbose = FALSE)

For the SnIPRE-like analysis, we model the counts of each type of mutation (Pn, Ps, Dn, Ds) in each gene in each organism as a poisson response variable. Following Eilertson et al (2012), we set the fixed effects to the length of the gene, the type of mutation (by fitting either a nonsynonymous or divergence effect), and the interaction between nonsynonymous and divergence effects. Because we are interested in estimating effects of certain pathways across species, we also fit nonsynonymous, divergence and nonsynonymous-by-divergence effects separately for each gene class and organism with the term nonsynonymous+divergence+nonsynonymous:divergence):(organism+piRNA+siRNA+miRNA+viRNA). The fixed effects portion of the model looks as follows:

summary(model.3A)

##
## Iterations = 3001:12991
## Thinning interval = 10
## Sample size = 1000
##
## DIC: 9979.245
##
## G-structure: ~us(1 + nonsynonymous + divergence + nonsynonymous:divergence):gene
##
## post.mean
## (Intercept):(Intercept).gene 0.0300609
## nonsynonymous:(Intercept).gene 0.0092904
## divergence:(Intercept).gene -0.0062428
## nonsynonymous:divergence:(Intercept).gene -0.0028054
## (Intercept):nonsynonymous.gene 0.0092904
## nonsynonymous:nonsynonymous.gene 0.7257428
## divergence:nonsynonymous.gene 0.0679281
## nonsynonymous:divergence:nonsynonymous.gene -0.0110024
## (Intercept):divergence.gene -0.0062428
## nonsynonymous:divergence.gene 0.0679281
## divergence:divergence.gene 0.0397815
## nonsynonymous:divergence:divergence.gene 0.0006943
## (Intercept):nonsynonymous:divergence.gene -0.0028054
## nonsynonymous:nonsynonymous:divergence.gene -0.0110024
## divergence:nonsynonymous:divergence.gene 0.0006943
## nonsynonymous:divergence:nonsynonymous:divergence.gene 0.0263167
## l-95% CI u-95% CI
## (Intercept):(Intercept).gene 2.216e-07 0.09298
## nonsynonymous:(Intercept).gene -9.516e-02 0.09389
## divergence:(Intercept).gene -4.354e-02 0.01857
## nonsynonymous:divergence:(Intercept).gene -3.070e-02 0.01703
## (Intercept):nonsynonymous.gene -9.516e-02 0.09389
## nonsynonymous:nonsynonymous.gene 5.079e-01 0.99459
## divergence:nonsynonymous.gene -1.419e-02 0.17282
## nonsynonymous:divergence:nonsynonymous.gene -9.904e-02 0.06650
## (Intercept):divergence.gene -4.354e-02 0.01857
## nonsynonymous:divergence.gene -1.419e-02 0.17282
## divergence:divergence.gene 8.076e-08 0.08962
## nonsynonymous:divergence:divergence.gene -2.709e-02 0.02161
## (Intercept):nonsynonymous:divergence.gene -3.070e-02 0.01703
## nonsynonymous:nonsynonymous:divergence.gene -9.904e-02 0.06650
## divergence:nonsynonymous:divergence.gene -2.709e-02 0.02161
## nonsynonymous:divergence:nonsynonymous:divergence.gene 3.772e-09 0.08581
## eff.samp
## (Intercept):(Intercept).gene 75.05
## nonsynonymous:(Intercept).gene 72.18
## divergence:(Intercept).gene 56.54
## nonsynonymous:divergence:(Intercept).gene 68.75
## (Intercept):nonsynonymous.gene 72.18
## nonsynonymous:nonsynonymous.gene 110.97
## divergence:nonsynonymous.gene 80.44
## nonsynonymous:divergence:nonsynonymous.gene 20.63
## (Intercept):divergence.gene 56.54
## nonsynonymous:divergence.gene 80.44
## divergence:divergence.gene 143.92
## nonsynonymous:divergence:divergence.gene 75.55
## (Intercept):nonsynonymous:divergence.gene 68.75
## nonsynonymous:nonsynonymous:divergence.gene 20.63
## divergence:nonsynonymous:divergence.gene 75.55
## nonsynonymous:divergence:nonsynonymous:divergence.gene 12.38
##
## R-structure: ~us(nfac):organism:gene:Duplicate
##
## post.mean l-95% CI u-95% CI
## nfac0 0:nfac0 0.organism:gene:Duplicate 0.61205 0.494162 0.73623
## nfac0 1:nfac0 0.organism:gene:Duplicate 0.40907 0.300592 0.52785
## nfac1 0:nfac0 0.organism:gene:Duplicate 0.07070 0.027120 0.12159
## nfac1 1:nfac0 0.organism:gene:Duplicate -0.04874 -0.134091 0.04111
## nfac0 0:nfac0 1.organism:gene:Duplicate 0.40907 0.300592 0.52785
## nfac0 1:nfac0 1.organism:gene:Duplicate 0.59946 0.457727 0.75343
## nfac1 0:nfac0 1.organism:gene:Duplicate 0.06481 0.008542 0.11479
## nfac1 1:nfac0 1.organism:gene:Duplicate 0.25187 0.146510 0.37272
## nfac0 0:nfac1 0.organism:gene:Duplicate 0.07070 0.027120 0.12159
## nfac0 1:nfac1 0.organism:gene:Duplicate 0.06481 0.008542 0.11479
## nfac1 0:nfac1 0.organism:gene:Duplicate 0.11693 0.079694 0.15503
## nfac1 1:nfac1 0.organism:gene:Duplicate 0.10049 0.050778 0.14859
## nfac0 0:nfac1 1.organism:gene:Duplicate -0.04874 -0.134091 0.04111
## nfac0 1:nfac1 1.organism:gene:Duplicate 0.25187 0.146510 0.37272
## nfac1 0:nfac1 1.organism:gene:Duplicate 0.10049 0.050778 0.14859
## nfac1 1:nfac1 1.organism:gene:Duplicate 0.42194 0.308206 0.54007
## eff.samp
## nfac0 0:nfac0 0.organism:gene:Duplicate 230.4
## nfac0 1:nfac0 0.organism:gene:Duplicate 170.7
## nfac1 0:nfac0 0.organism:gene:Duplicate 122.4
## nfac1 1:nfac0 0.organism:gene:Duplicate 153.7
## nfac0 0:nfac0 1.organism:gene:Duplicate 170.7
## nfac0 1:nfac0 1.organism:gene:Duplicate 178.2
## nfac1 0:nfac0 1.organism:gene:Duplicate 115.4
## nfac1 1:nfac0 1.organism:gene:Duplicate 184.9
## nfac0 0:nfac1 0.organism:gene:Duplicate 122.4
## nfac0 1:nfac1 0.organism:gene:Duplicate 115.4
## nfac1 0:nfac1 0.organism:gene:Duplicate 168.3
## nfac1 1:nfac1 0.organism:gene:Duplicate 321.7
## nfac0 0:nfac1 1.organism:gene:Duplicate 153.7
## nfac0 1:nfac1 1.organism:gene:Duplicate 184.9
## nfac1 0:nfac1 1.organism:gene:Duplicate 321.7
## nfac1 1:nfac1 1.organism:gene:Duplicate 242.8
##
## Location effects: count ~ log(length) + nonsynonymous + divergence + nonsynonymous:divergence + organism + (nonsynonymous + divergence + nonsynonymous:divergence):(organism + piRNA + viRNA + miRNA + siRNA)
##
## post.mean l-95% CI u-95% CI
## (Intercept) -4.46904 -4.97306 -3.94758
## log(length) 0.97623 0.91561 1.04389
## nonsynonymous -1.49970 -1.77328 -1.24692
## divergence 1.21542 1.00947 1.42464
## organismanopheles 0.21142 -0.08762 0.44189
## organismapis -1.81890 -2.10749 -1.52871
## organismbombyx 0.40217 0.14216 0.69221
## organismdpse -0.43007 -0.70159 -0.18840
## organismheliconius 0.05955 -0.21181 0.34851
## nonsynonymous:divergence 0.25602 0.01122 0.46658
## nonsynonymous:organismanopheles -0.46541 -0.75293 -0.17766
## nonsynonymous:organismapis -0.40197 -0.77895 -0.04547
## nonsynonymous:organismbombyx -0.24661 -0.58073 0.06554
## nonsynonymous:organismdpse 0.34119 0.03611 0.64421
## nonsynonymous:organismheliconius 0.01839 -0.27399 0.31943
## nonsynonymous:piRNA 0.54011 0.15190 0.93668
## nonsynonymous:viRNA 0.58109 -0.60559 1.64163
## nonsynonymous:miRNA -0.44753 -1.18277 0.25110
## nonsynonymous:siRNA -0.82456 -1.74869 0.04430
## divergence:organismanopheles -0.95889 -1.23915 -0.67998
## divergence:organismapis 1.06272 0.77986 1.36073
## divergence:organismbombyx 0.27959 -0.01646 0.53432
## divergence:organismdpse -0.76135 -1.03303 -0.50080
## divergence:organismheliconius -1.06374 -1.31553 -0.76169
## divergence:piRNA 0.08859 -0.04536 0.21826
## divergence:viRNA 0.13431 -0.24487 0.45353
## divergence:miRNA -0.02860 -0.24507 0.18153
## divergence:siRNA -0.04164 -0.30738 0.20942
## nonsynonymous:divergence:organismanopheles 0.05467 -0.17126 0.27527
## nonsynonymous:divergence:organismapis -0.35464 -0.63657 0.02153
## nonsynonymous:divergence:organismbombyx 0.06181 -0.22513 0.33049
## nonsynonymous:divergence:organismdpse 0.39663 0.17378 0.65118
## nonsynonymous:divergence:organismheliconius -0.55927 -0.78868 -0.34668
## nonsynonymous:divergence:piRNA 0.36223 0.13452 0.59720
## nonsynonymous:divergence:viRNA 0.90780 0.50794 1.33614
## nonsynonymous:divergence:miRNA 0.26052 -0.06620 0.51761
## nonsynonymous:divergence:siRNA 0.78372 0.18102 1.45044
## eff.samp pMCMC
## (Intercept) 101.086 <0.001 ***
## log(length) 82.580 <0.001 ***
## nonsynonymous 56.027 <0.001 ***
## divergence 403.392 <0.001 ***
## organismanopheles 723.666 0.126
## organismapis 264.677 <0.001 ***
## organismbombyx 514.172 0.004 **
## organismdpse 311.035 0.002 **
## organismheliconius 706.023 0.640
## nonsynonymous:divergence 11.749 0.038 *
## nonsynonymous:organismanopheles 150.165 <0.001 ***
## nonsynonymous:organismapis 20.804 0.020 *
## nonsynonymous:organismbombyx 60.972 0.140
## nonsynonymous:organismdpse 36.646 0.038 *
## nonsynonymous:organismheliconius 147.820 0.888
## nonsynonymous:piRNA 211.155 0.008 **
## nonsynonymous:viRNA 294.525 0.310
## nonsynonymous:miRNA 326.843 0.236
## nonsynonymous:siRNA 16.762 0.082 .
## divergence:organismanopheles 529.568 <0.001 ***
## divergence:organismapis 254.734 <0.001 ***
## divergence:organismbombyx 345.638 0.052 .
## divergence:organismdpse 291.953 <0.001 ***
## divergence:organismheliconius 654.453 <0.001 ***
## divergence:piRNA 437.990 0.178
## divergence:viRNA 321.484 0.422
## divergence:miRNA 689.476 0.764
## divergence:siRNA 574.703 0.752
## nonsynonymous:divergence:organismanopheles 39.134 0.680
## nonsynonymous:divergence:organismapis 8.341 0.080 .
## nonsynonymous:divergence:organismbombyx 19.681 0.644
## nonsynonymous:divergence:organismdpse 18.507 <0.001 ***
## nonsynonymous:divergence:organismheliconius 42.853 <0.001 ***
## nonsynonymous:divergence:piRNA 13.101 0.004 **
## nonsynonymous:divergence:viRNA 36.579 <0.001 ***
## nonsynonymous:divergence:miRNA 17.685 0.092 .
## nonsynonymous:divergence:siRNA 5.187 0.016 *
## ---
## Signif. codes: 0 '***' 0.001 '**' 0.01 '*' 0.05 '.' 0.1 ' ' 1

The model output shows organisms (e.g. nonsynonymous:divergence:organismapis effect) and subpathways (e.g. nonsynonymous:divergence:viRNA effect) differ in their genome-wide level of positive selection. We test whether a homologue has an increased selection effect (e.g. Figure 4, Figure S4) by comparing the posterior distributions of the selection effect for control genes and the homologues. For example:

ago2.selection.effect <- model.3A$Sol[,"nonsynonymous:divergence"] +
 model.3A$Sol[,"nonsynonymous:divergence:viRNA"] +
 model.3A$Sol[,"nonsynonymous:divergence.gene.ago2"]

control.selection.effect <- model.3A$Sol[,"nonsynonymous:divergence"]

control.minus.ago2.posterior <- control.selection.effect - ago2.selection.effect

iterations.less.than.zero <- length(which(control.minus.ago2.posterior > 0))
total.chain.length <- length(control.minus.ago2.posterior)
pMCMC <- iterations.less.than.zero/total.chain.length
pMCMC*2 #Multiply by 2 to make a two tailed test

## [1] 0

We conclude that the Ago2 selection effect is greater than control genes (MCMCp < 0.001).

In addition to homologue-specific random effects, we include gene-specific random effects in the SnIPRE model. The homologue-specific random effects are coded as "random=~us(1 + nonsynonymous+divergence+nonsynonymous:divergence):gene", very similar to the random effect structure of Eilertson et al (2012), except we have an average of 24 observations per gene (6 species by 4 types of mutation). Instead of using the per-gene random effect structure from Eilertson et al (2012), we reparameterise the model so that residuals for each class of mutation would be estimated for each gene, and (by specifying pl=TRUE), the posterior distribution of each of these residuals stored. We code the different classes of mutation in the nfac column, where "0 0" denotes synonymous polymorphism, "0 1" denotes nonsynonymous polymorphism, and so on. The unstructured covariance matrix between mutation classes (us(nfac)) is estimated for each gene (organism:gene:Duplicate), from which we extract the gene-level selection effect. The posterior distributions of these residuals are saved in the model.3A$Liab data structure, with columns in the same order as the rows in our original data table:

nrow(dat) == ncol(data.frame(model.3A$Liab))

## [1] TRUE

To extract the gene specific selection effect from each residual, we create the mapping matrix (X), the model matrix for all nonsynonymous:divergence effects (X.matrix, a template for which fixed effects link with the rows of data), the columns of the model.3A fixed effects which match X.matrix (X.model.hit), and a list of unique genes (unique.genes):

X<-rbind(c(1,0,0,0), c(0,0,1,0), c(0,1,0,0), c(0,1,1,1))
X.model<-model.matrix(~nonsynonymous:divergence-1+(nonsynonymous:divergence):(piRNA+siRNA+miRNA+viRNA+gene), data=dat)
colnames(X.model) <- gsub(colnames(X.model), pattern= ":gene", replacement = "\\.gene\\.")
X.model.hit<-match(colnames(X.model), colnames(model.3A$Sol))
unique.genes<-unique(dat$gene.id)
residuals.transform<-model.3A$Liab
X.fixed.random.effects<-model.3A$Liab

We loop through rows of the stored latent variables (model.3A$Liab), with each row being an iteration of the MCMC chain, and subtract the fixed and random effects corresponding to each data point ((model.3A$X%*%model.3A$Sol[i,1:ncol(model.3A$X)])) from the ωA estimate (model.3A$Z%*%model.3A$Sol[i,(ncol(model.3A$X)+1):ncol(model.3A$Sol)])), resulting in residuals for each gene in each species. Then, for each gene (gene.id - a combination of gene name, organism, and duplicate) in each iteration, we map the residuals onto the design matrix X to solve for the random effects of each observation. (residuals.transform). Finally, we obtain the posterior distribution of the selection effect for a particular gene (selection.effects) by adding the random nonsynonymous:divergence:gene effect (residuals.transform) to the fixed and random nonsynonymous:divergence effects (X.fixed.random.effects).

for(i in 1:nrow(model.3A$Liab)){
 residuals<-model.3A$Liab[i,]-(model.3A$X%*%model.3A$Sol[i,1:ncol(model.3A$X)])@x -
 (model.3A$Z%*%model.3A$Sol[i,(ncol(model.3A$X)+1):ncol(model.3A$Sol)])@x #Subtract the fixed pathway effects and random homologue effects from the latent variables
 # residuals for each observation at iteration i
 for(j in 1:length(unique.genes)){
 hits<-which(dat$gene.id==unique.genes[j])
 # find positions of residuals gene j
 if(length(hits)==4){
 beta<-solve(X,residuals[hits]) # Map the residuals from the difference between latent variables and fixed effects onto design matrix
 residuals.transform[i,hits]<-beta
 # solve for the (random) b effects for each observation
 X.fixed.random.effects[i,hits]<-c(X.model[hits,]%*%model.3A$Sol[i,X.model.hit])
 # get nonsynonymous+divergence predictions for each obseravtion
 }
 }
}
selection.effects<-data.frame(X.fixed.random.effects+residuals.transform)
selection.effects <- selection.effects[,(dat$nfac == "1 1") & (dat$RNAi == 1)]
colnames(selection.effects) <- dat$gene.id[(dat$nfac == "1 1") & (dat$RNAi == 1)]
head(selection.effects)[,1:5]

## anopheles ago1 A apis ago1 A bombyx ago1 A dmel ago1 A dpse ago1 A
## 1 1.4193757 0.7722761 0.5842877 0.08680539 0.3625202
## 2 0.8455055 0.6456496 0.4544315 0.23100181 0.1364505
## 3 0.4975981 0.5436095 0.2534048 -0.33675612 -0.6107161
## 4 1.4048516 0.6310795 0.1799840 0.83226188 -0.5621510
## 5 1.2629835 0.3232525 -0.3350957 0.34226755 -0.1326866
## 6 0.7548817 0.3769485 -0.4292804 0.56985419 0.1456308

We calculate a "species-corrected" selection effect where nonsynonymous:divergence:organism effects are excluded in order to visualise differences between subpathways (e.g. Figure 3). We add these effects later when assessing positive selection in individual genes within a species. Also, in this example, we have only solved for the gene-level nonsynonymous:divergence random effects, however, the other gene-level effects could be obtained in a similar way.

SnIPRE was originally intended to identify genes in a single organism which shows signs of elevated positive selection. To do this, we add the organism specific selection effect to the "species-corrected" selection effect we have already obtained, and ask whether the selection effect overlaps zero. For example, to estimate the selection effect for the genes in Apis mellifera, we add the nonsynonymous:divergence:apis posterior to the columns of selection.effects which belong to Apis.

apis <- model.3A$Sol[,"nonsynonymous:divergence:organismapis"]
selection.effects.apis <- selection.effects[,grep("apis",colnames(selection.effects))] + apis

Then, using HPDinterval() and colMeans(), we can get the selection effect for each gene, along with the upper and lower 95% highest posterior density intervals.

selection.effects.apis.summary <- data.frame(HPDinterval(as.mcmc(selection.effects.apis)))
selection.effects.apis.summary <- cbind(selection.effects.apis.summary,data.frame(selectioneffect=c(as.vector(colMeans(selection.effects.apis)))))
head(selection.effects.apis.summary)

## lower upper selectioneffect
## apis ago1 A -1.0316050 1.2296024 0.16360626
## apis ago2 A 0.8680883 2.3909126 1.64137753
## apis ago3 A -0.4233092 1.2884881 0.35796870
## apis armi A -0.6455877 0.7797752 0.06855869
## apis ars2 A -1.0879748 0.6938276 -0.13471034
## apis arx A -1.0893511 1.0259998 -0.06135972

Finally, we identify genes with significantly positive selection effects.

selection.effects.apis.significant <- selection.effects.apis.summary[selection.effects.apis.summary$lower > 0,]
print(selection.effects.apis.significant)

## lower upper selectioneffect
## apis ago2 A 0.868088349 2.390913 1.6413775
## apis piwi A 0.350946926 1.734872 1.0338514
## apis dcr2 A 0.382093697 1.785551 1.0686899
## apis hen1 A 0.125590039 1.452687 0.7121331
## apis r2d2 A 0.007973288 2.385391 1.2971375
## apis tud A 0.280620893 1.627606 0.9377881
## apis vas A 0.615037761 2.525881 1.5089618

Model 3B: SnIPRE-like analysis, with piRNA split into biogenesis factors, effectors, and transcriptional silencing

prior.3B <-list(R=list(V=diag(4), nu=0.002),
 G=list(G1=list(V=diag(4), nu=4, alpha.mu=rep(0,4), alpha.V=diag(4)*1000)))
model.3B <- MCMCglmm(count~log(length)+nonsynonymous + divergence+nonsynonymous:divergence+organism+
 (nonsynonymous+divergence+nonsynonymous:divergence):(organism + effector + biogenesis + transcriptional + viRNA + miRNA + siRNA),
 random=~us(1 + nonsynonymous + divergence + nonsynonymous:divergence):gene,
 rcov=~us(nfac):organism:gene:Duplicate, family="poisson",
 data=dat, pr = TRUE, pl=TRUE, prior = prior.3B, verbose = FALSE)

We also fit the SnIPRE model (Model 3A) with the piRNA pathway split into different functional categories, akin to the Model 1B and 1C. We only used this model to estimate the selection effects associated with the piRNA categories (transcriptional silencing, effectors, and biogenesis machinery).

Model 3C: SnIPRE-like analysis, without subpathway as a fixed effect

prior.3C <- list(R=list(V=diag(4), nu=0.002), G=list(G1=list(V=diag(4), nu=4, alpha.mu=rep(0,4), alpha.V=diag(4)*1000)))
model.3C <- MCMCglmm(count~log(length)+nonsynonymous + divergence+nonsynonymous:divergence+organism+
 (nonsynonymous+divergence+nonsynonymous:divergence):(organism),
 random=~us(1 + nonsynonymous + divergence + nonsynonymous:divergence):gene,
 rcov=~us(nfac):organism:gene:Duplicate, family="poisson",
 data=dat, pr = TRUE, pl=TRUE, prior = prior.3C, verbose = FALSE)

Finally, we fit the SniPRE model (Model 5A) without assuming genes belong to any particular subpathway,similar to the difference between Model 2A and 2B. Selection effects were then calculated in the same way, excluding the addition of a subpathway fixed effect.

S2 Text: Coalescent simulations

To assess significance in the SweeD analyses, we used ms (Hudson, 2002) to perform 1000 coalescent simulations for each gene region of interest in each species, given the observed number of segregating sites, reported recombination rate, and a previously published estimate of the demographic history of that species. When population scaled recombination rate estimates were not available, we used estimates of *N_e_* to scale per-base rate estimates. Although the details of the demographic scenarios we modelled are unlikely to impact substantially upon our qualitative comparisons of between sweep frequency in different types of gene, we attempted to use null models consistent with the published literature. The demographic scenarios modelled for each species are illustrated in Figure S1. For *D. melanogaster*, recombination rates from the *Drosophila* recombination rate calculator were used with a constant *N_e_* for African populations of 1.15x10^6^ (Charlesworth, 2009). Some genes (*ael, AGO3, pasha,* and *Rm62*) are reported to lie in areas with zero recombination (Fiston-Lavier, et al., 2010), so we set the recombination rate in these genes at the lowest non-zero rate observed. For *D. pseudoobscura,* we simulated a population expansion (Haddrill, et al., 2010; Larracuente & Clark, 2014), and used the population scaled rates of recombination and gene conversion from Larracuente and Clark (2014). For *Anopheles gambiae,* we used demographic history parameters from Crawford and Lazzaro et al (2010) for the Cameroon population, and the recombination rates for each individual chromosome arm (1 cM/Mb for the X, 1.3 cM/Mb for 3L and 2R, 1.6 cM/Mb for 3R, and 2 cM/Mb for 2L) from Pombi et al (2006) and Stump et al (2007). Effective population size (*N_e_*) was set to 2.4x10^6^ estimated using the *D. melanogaster* mutation rate of Keightley et al (2014) and the Watterson’s theta (θ_W_) estimate in Crawford and Lazzaro (2010). For *H. melpomene,* we simulated three Costa Rican populations corresponding to *H. melpomene, H. cydno, and H. pachinus,* using the migration rates provided in Table 2 of Kronforst et al (2006). We used a constant recombination rate of 7.51 cM/Mb across the entire genome with an *N_e_* of 2.1x10^6^ for *H. melpomene*, 3.3x10^6^ for cydno, and 2.7x10^6^ for *H. pachinus*. For *B. mandarina,* we modelled the “gene-flow at bottleneck” scenario (Yang et al, 2014), with an *N_e_* of 500,000 for *B. mandarina* and 73,000 for *B. mori*, and a recombination rate of 2.97 cM/Mb (Yamamoto et al, 2008; Yang et al, 2014). For *A. mellifera,* four subpopulations were modelled using *N_e_* values in Table 1 of Wallberg et al (2014), following Figure 1F in Wallberg et al (2014) when modelling past subpopulation size changes. These subpopulations share migrants, and migration rates were estimated based on *F*_ST_ values between subpopulations reported in Whitfield et al (2006). A recombination rate of 19 cM/Mb is assumed to be constant across the genome (Beye et al, 2006). For *C. briggsae*, coalescent simulations and SweeD analyses were carried out on the 25 “tropical” samples in order to avoid modelling complicated demographic scenarios. These are expected to have an effective population size of 60,000, and to have undergone a recent bottleneck 0.916 *N_e_* generations in the past (Cutter et al, 2006; Denver et al, 2009), assuming a 60-day generation time (Barrière & Félix, 2005). We used recombination rates for *C. briggsae* from Ross et al (2011), which are estimated to be 9.97 x 10^-8^ per bp per generation in autosomes and 4.6 x 10^-8^ per bp per generation on the X chromosome (Ross et al, 2011). Finally, for *P. pacificus,* four subpopulations were modelled corresponding to clade A1, A2, C, and 9 individuals whose clade was unknown (Rödelsperger, et al., 2014) which coalesced 0.849 *N_e_* generations in the past (McGaughran et al, 2013). *N_e_* was estimated by calculating θ_W_ for each contig and assuming a mutation rate of2x10^-9^ (Weller et al, 2014).To minimise differences between the real data and simulations, sites were randomly chosen to be folded, ancestrally invariant, or fixed for a derived substitution, in each case matching the numbers observed in the real data before the SweeD analysis.

**Supplementary Materials References**

Barrière, Antoine, and Marie-Anne Félix. 2005. "High local genetic diversity and low outcrossing rate in Caenorhabditis elegans natural populations." *Current biology : CB* 15 (13): 1176-84.

Beye, Martin, Irene Gattermeier, Martin Hasselmann, Tanja Gempe, Morten Schioett, John F Baines, David Schlipalius, et al. 2006. "Exceptionally high levels of recombination across the honey bee genome." *Genome research* 16 (11): 1339-44.

Boeke, J., I. Bag, M. J. Ramaiah, I. Vetter, E. Kremmer et al., 2011 The RNA Helicase Rm62 Cooperates with SU(VAR)3-9 to Re-Silence Active Transcription in Drosophila melanogaster (L. Tora, Ed.). PLoS One 6: e20761.

Brennecke, Julius, Alexei A Aravin, Alexander Stark, Monica Dus, Manolis Kellis, Ravi Sachidanandam, and Gregory J Hannon. 2007. "Discrete small RNA-generating loci as master regulators of transposon activity in Drosophila." *Cell* 128 (6): 1089-103.

Caudy, Amy A, Mike Myers, Gregory J Hannon, and Scott M Hammond. 2002. "Fragile X-related protein and VIG associate with the RNA interference machinery." *Genes & development* (Cold Spring Harbor Laboratory Press) 16 (19): 2491-6.

Caudy, Amy A., René F. Ketting, Scott M. Hammond, Ahmet M. Denli, Anja M. P. Bathoorn, Bastiaan B. J. Tops, Jose M. Silva, Mike M. Myers, Gregory J. Hannon, and Ronald H. A. Plasterk. 2003. "A micrococcal nuclease homologue in RNAi effector complexes." *Nature* (Nature Publishing Group) 425 (6956): 411-414.

Charlesworth, Brian. 2009. "Fundamental concepts in genetics: effective population size and patterns of molecular evolution and variation." *Nature reviews. Genetics* (Nature Publishing Group) 10 (3): 195-205.

Crawford, Jacob E, and Brian P Lazzaro. 2010. "The demographic histories of the M and S molecular forms of Anopheles gambiae s.s." *Molecular biology and evolution* 27 (8): 1739-44.

Csink, A K, R Linsk, and J A Birchler. 1994. "The Lighten up (Lip) gene of Drosophila melanogaster, a modifier of retroelement expression, position effect variegation and white locus insertion alleles." *Genetics* 138 (1): 153-63.

Cutter, Asher D, Marie-Anne Félix, Antoine Barrière, and Deborah Charlesworth. 2006. "Patterns of nucleotide polymorphism distinguish temperate and tropical wild isolates of Caenorhabditis briggsae." *Genetics* 173 (4): 2021-31.

Czech, Benjamin, Jonathan B Preall, Jon McGinn, and Gregory J Hannon. 2013. "A transcriptome-wide RNAi screen in the Drosophila ovary reveals factors of the germline piRNA pathway." *Molecular cell* 50 (5): 749-61.

Denver, Dee R, Peter C Dolan, Larry J Wilhelm, Way Sung, J Ignacio Lucas-Lledó, Dana K Howe, Samantha C Lewis, et al. 2009. "A genome-wide view of Caenorhabditis elegans base-substitution mutation processes." *Proceedings of the National Academy of Sciences of the United States of America* 106 (38): 16310-4.

Dönertas, Derya, Grzegorz Sienski, and Julius Brennecke. 2013. "Drosophila Gtsf1 is an essential component of the Piwi-mediated transcriptional silencing complex." *Genes & development* (Cold Spring Harbor Laboratory Press) 27 (15): 1693-705.

Eilertson, Kirsten E, James G Booth, and Carlos D Bustamante. 2012. "SnIPRE: selection inference using a Poisson random effects model." *PLoS computational biology* (Public Library of Science) 8 (12): e1002806.

Fiston-Lavier, Anna-Sophie, Nadia D Singh, Mikhail Lipatov, and Dmitri A Petrov. 2010. "Drosophila melanogaster recombination rate calculator." *Gene* 463 (1-2): 18-20.

Gruber, Joshua J., D. Steven Zatechka, Leah R. Sabin, Jeongsik Yong, Julian J. Lum, Mei Kong, Wei-Xing Zong, et al. 2009. "Ars2 Links the Nuclear Cap-Binding Complex to RNA Interference and Cell Proliferation." *Cell* 138 (2): 328-339.

Gunawardane, Lalith S., Kuniaki Saito, Kazumichi M. Nishida, Keita Miyoshi, Yoshinori Kawamura, Tomoko Nagami, Haruhiko Siomi, and Mikiko C. Siomi. 2007. "A Slicer-Mediated Mechanism for Repeat-Associated siRNA 5' End Formation in Drosophila." *Science* 315 (5818).

Haase, Astrid D, Silvia Fenoglio, Felix Muerdter, Paloma M Guzzardo, Benjamin Czech, Darryl J Pappin, Caifu Chen, Assaf Gordon, and Gregory J Hannon. 2010. "Probing the initiation and effector phases of the somatic piRNA pathway in Drosophila." *Genes & development* (Cold Spring Harbor Laboratory Press) 24 (22): 2499-504.

Haddrill, Penelope R, Laurence Loewe, and Brian Charlesworth. 2010. "Estimating the parameters of selection on nonsynonymous mutations in Drosophila pseudoobscura and D. miranda." *Genetics* 185 (4): 1381-96.

Han, Bo W, Wei Wang, Chengjian Li, Zhiping Weng, and Phillip D Zamore. 2015. "piRNA-guided transposon cleavage initiates Zucchini-dependent, phased piRNA production." *Science (New York, N.Y.)* 348 (6236): 817-21.

Handler, Dominik, Katharina Meixner, Manfred Pizka, Kathrin Lauss, Christopher Schmied, Franz Sebastian Gruber, and Julius Brennecke. 2013. "The genetic makeup of the Drosophila piRNA pathway." *Molecular cell* 50 (5): 762-77.

Horwich, Michael D., Chengjian Li, Christian Matranga, Vasily Vagin, Gwen Farley, Peng Wang, and Phillip D. Zamore. 2007. "The Drosophila RNA Methyltransferase, DmHen1, Modifies Germline piRNAs and Single-Stranded siRNAs in RISC." 1265-1272.

Hudson, R. R. 2002. "Generating samples under a Wright-Fisher neutral model of genetic variation." *Bioinformatics* (Oxford University Press) 18 (2): 337-338.

Ishizuka, Akira, Mikiko C Siomi, and Haruhiko Siomi. 2002. "A Drosophila fragile X protein interacts with components of RNAi and ribosomal proteins." *Genes & development* (Cold Spring Harbor Laboratory Press) 16 (19): 2497-508.

Jiang, Feng, Xuecheng Ye, Xiang Liu, Lauren Fincher, Dennis McKearin, and Qinghua Liu. 2005. "Dicer-1 and R3D1-L catalyze microRNA maturation in Drosophila." *Genes & development* (Cold Spring Harbor Laboratory Press) 19 (14): 1674-9.

Keightley, Peter D, Rob W Ness, Daniel L Halligan, and Penelope R Haddrill. 2014. "Estimation of the spontaneous mutation rate per nucleotide site in a Drosophila melanogaster full-sib family." *Genetics* 196 (1): 313-20.

Kirino, Yohei, Namwoo Kim, Mariàngels de Planell-Saguer, Eugene Khandros, Stephanie Chiorean, Peter S. Klein, Isidore Rigoutsos, Thomas A. Jongens, and Zissimos Mourelatos. 2009. "Arginine methylation of Piwi proteins catalysed by dPRMT5 is required for Ago3 and Aub stability." *Nature Cell Biology* (Nature Publishing Group) 11 (5): 652-658.

Klattenhoff, Carla, Hualin Xi, Chengjian Li, Soohyun Lee, Jia Xu, Jaspreet S. Khurana, Fan Zhang, et al. 2009. "The Drosophila HP1 Homolog Rhino Is Required for Transposon Silencing and piRNA Production by Dual-Strand Clusters." *Cell* 138 (6): 1137-1149.

Koch, Carmen M., Mona Honemann-Capito, Diane Egger-Adam, and Andreas Wodarz. 2009. "Windei, the Drosophila Homolog of mAM/MCAF1, Is an Essential Cofactor of the H3K9 Methyl Transferase dSETDB1/Eggless in Germ Line Development." Edited by Asifa Akhtar. *PLoS Genetics* 5 (9): e1000644.

Kronforst, Marcus R., Laura G. Young, Lauren M. Blume, and Lawrence E. Gilbert. 2006. "Multilocus analyses of admixture and introgression among hybridizing Heliconius butterflies." *Evolution* 60 (6): 1254-1268.

Larracuente, Amanda M, and Andrew G Clark. 2014. "Recent selection on the Y-to-dot translocation in Drosophila pseudoobscura." *Molecular biology and evolution* 31 (4): 846-56.

Lee, Yoontae, Chiyoung Ahn, Jinju Han, Hyounjeong Choi, Jaekwang Kim, Jeongbin Yim, Junho Lee, et al. 2003. "The nuclear RNase III Drosha initiates microRNA processing." *Nature* (Nature Publishing Group) 425 (6956): 415-419.

Lewis, Samuel H, Heli Salmela, and Darren J Obbard. 2016. "Duplication and Diversification of Dipteran Argonaute Genes, and the Evolutionary Divergence of Piwi and Aubergine." *Genome biology and evolution* 8 (3): 507-18.

Lewis, Samuel H., Claire L. Webster, Heli Salmela, and Darren J. Obbard. 2016. "Repeated Duplication of Argonaute2 Is Associated with Strong Selection and Testis Specialization in Drosophila." *Genetics* 204 (2).

Liu, Qinghua, Tim A. Rand, Savitha Kalidas, Fenghe Du, Hyun-Eui Kim, Dean P. Smith, and Xiaodong Wang. 2003. "R2D2, a Bridge Between the Initiation and Effector Steps of the Drosophila RNAi Pathway." *Science* 301 (5641).

Liu, Y., X. Ye, F. Jiang, C. Liang, D. Chen, J. Peng, L. N. Kinch, N. V. Grishin, and Q. Liu. 2009. "C3PO, an Endoribonuclease That Promotes RNAi by Facilitating RISC Activation." *Science* 325 (5941): 750-753.

Lo, Pang-Kuo, Yi-Chun Huang, John S. Poulton, Nicholas Leake, William H. Palmer, Daniel Vera, Gengqiang Xie, Stephen Klusza, and Wu-Min Deng. 2016. "RNA helicase Belle/DDX3 regulates transgene expression in Drosophila." *Developmental Biology* 412 (1): 57-70.

Malone, Colin D., Julius Brennecke, Monica Dus, Alexander Stark, W. Richard McCombie, Ravi Sachidanandam, and Gregory J. Hannon. 2009. "Specialized piRNA Pathways Act in Germline and Somatic Tissues of the Drosophila Ovary." *Cell* 137 (3): 522-535.

McGaughran, Angela, Katy Morgan, and Ralf J Sommer. 2013. "Unraveling the evolutionary history of the nematode Pristionchus pacificus: from lineage diversification to island colonization." *Ecology and evolution* 3 (3): 667-75.

Mohn, Fabio, Dominik Handler, and Julius Brennecke. 2015. "piRNA-guided slicing specifies transcripts for Zucchini-dependent, phased piRNA biogenesis." *Science (New York, N.Y.)* 348 (6236): 812-7.

Mohn, Fabio, Grzegorz Sienski, Dominik Handler, and Julius Brennecke. 2014. "The Rhino-Deadlock-Cutoff Complex Licenses Noncanonical Transcription of Dual-Strand piRNA Clusters in Drosophila." *Cell* 157 (6): 1364-1379.

Muerdter, Felix, Paloma M Guzzardo, Jesse Gillis, Yicheng Luo, Yang Yu, Caifu Chen, Richard Fekete, and Gregory J Hannon. 2013. "A genome-wide RNAi screen draws a genetic framework for transposon control and primary piRNA biogenesis in Drosophila." *Molecular cell* 50 (5): 736-48.

Neumüller, Ralph A., Joerg Betschinger, Anja Fischer, Natascha Bushati, Ingrid Poernbacher, Karl Mechtler, Stephen M. Cohen, and Juergen A. Knoblich. 2008. "Mei-P26 regulates microRNAs and cell growth in the Drosophila ovarian stem cell lineage." *Nature* (Nature Publishing Group) 454 (7201): 241-245.

Nishida, Kazumichi M, Tomoko N Okada, Takeshi Kawamura, Toutai Mituyama, Yoshinori Kawamura, Sachi Inagaki, Haidong Huang, et al. 2009. "Functional involvement of Tudor and dPRMT5 in the piRNA processing pathway in Drosophila germlines." *The EMBO journal* (European Molecular Biology Organization) 28 (24): 3820-31.

Ohtani, Hitoshi, Yuka W Iwasaki, Aoi Shibuya, Haruhiko Siomi, Mikiko C Siomi, and Kuniaki Saito. 2013. "DmGTSF1 is necessary for Piwi-piRISC-mediated transcriptional transposon silencing in the Drosophila ovary." *Genes & development* 27 (15): 1656-61.

Okamura, Katsutomo, Akira Ishizuka, Haruhiko Siomi, and Mikiko C Siomi. 2004. "Distinct roles for Argonaute proteins in small RNA-directed RNA cleavage pathways." *Genes & development* (Cold Spring Harbor Laboratory Press) 18 (14): 1655-66.

Olivieri, Daniel, Kirsten-André Senti, Sailakshmi Subramanian, Ravi Sachidanandam, and Julius Brennecke. 2012. "The Cochaperone Shutdown Defines a Group of Biogenesis Factors Essential for All piRNA Populations in Drosophila." *Molecular Cell* 47 (6): 954-969.

Pane, Attilio, Peng Jiang, Dorothy Yanling Zhao, Mona Singh, and Trudi Schüpbach. 2011. "The Cutoff protein regulates piRNA cluster expression and piRNA production in the Drosophila germline." *The EMBO journal* 30 (22): 4601-15.

Parhad, S. S., S. Tu, Z. Weng, and W. E. Theurkauf, 2017 Adaptive Evolution Leads to Cross-Species Incompatibility in the piRNA Transposon Silencing Machinery. Dev. Cell 43: 60–70.e5.

Patil, Veena S, Amit Anand, Alisha Chakrabarti, and Toshie Kai. 2014. "The Tudor domain protein Tapas, a homolog of the vertebrate Tdrd7, functions in the piRNA pathway to regulate retrotransposons in germline of Drosophila melanogaster." *BMC Biology* 12 (1): 61.

Patil, Veena S., and Toshie Kai. 2010. "Repression of Retroelements in Drosophila Germline via piRNA Pathway by the Tudor Domain Protein Tejas." 724-730.

Pombi, Marco, Aram D Stump, Alessandra Della Torre, and Nora J Besansky. 2006. "Variation in recombination rate across the X chromosome of Anopheles gambiae." *The American journal of tropical medicine and hygiene* 75 (5): 901-3.

Preall, Jonathan B, Benjamin Czech, Paloma M Guzzardo, Felix Muerdter, and Gregory J Hannon. 2012. "shutdown is a component of the Drosophila piRNA biogenesis machinery." *RNA (New York, N.Y.)* (Cold Spring Harbor Laboratory Press) 18 (8): 1446-57.

Rangan, Prashanth, Colin D. Malone, Caryn Navarro, Sam P. Newbold, Patrick S. Hayes, Ravi Sachidanandam, Gregory J. Hannon, and Ruth Lehmann. 2011. "piRNA Production Requires Heterochromatin Formation in Drosophila." 1373-1379.

Rödelsperger, Christian, Richard A Neher, Andreas M Weller, Gabi Eberhardt, Hanh Witte, Werner E Mayer, Christoph Dieterich, and Ralf J Sommer. 2014. "Characterization of genetic diversity in the nematode Pristionchus pacificus from population-scale resequencing data." *Genetics* 196 (4): 1153-65.

Ross, Joseph A, Daniel C Koboldt, Julia E Staisch, Helen M Chamberlin, Bhagwati P Gupta, Raymond D Miller, Scott E Baird, and Eric S Haag. 2011. "Caenorhabditis briggsae recombinant inbred line genotypes reveal inter-strain incompatibility and the evolution of recombination." *PLoS genetics* 7 (7): e1002174.

Ryazansky, S. S., A. A. Kotov, M. V. Kibanov, N. V. Akulenko, A. P. Korbut et al., 2016 RNA helicase Spn-E is required to maintain Aub and AGO3 protein levels for piRNA silencing in the germline of Drosophila. Eur. J. Cell Biol. 95: 311–322.

Sabin, L. R., R. Zhou, J. J. Gruber, N. Lukinova, S. Bambina et al., 2009 Ars2 regulates both miRNA- and siRNA- dependent silencing and suppresses RNA virus infection in Drosophila. Cell 138: 340–51.

Saito, K., Y. Sakaguchi, T. Suzuki, T. Suzuki, H. Siomi, and M. C. Siomi. 2007. "Pimet, the Drosophila homolog of HEN1, mediates 2'-O-methylation of Piwi- interacting RNAs at their 3' ends." *Genes & Development* 21 (13): 1603-1608.

Saito, Kuniaki, Akira Ishizuka, Haruhiko Siomi, Mikiko C Siomi, EJ Sontheimer, and T Tuschl. 2005. "Processing of Pre-microRNAs by the Dicer-1–Loquacious Complex in Drosophila Cells." Edited by James C. Carrington. *PLoS Biology* (Public Library of Science) 3 (7): e235.

Saito, Kuniaki, Hirotsugu Ishizu, Miharu Komai, Hazuki Kotani, Yoshinori Kawamura, Kazumichi M Nishida, Haruhiko Siomi, and Mikiko C Siomi. 2010. "Roles for the Yb body components Armitage and Yb in primary piRNA biogenesis in Drosophila." *Genes & development* (Cold Spring Harbor Laboratory Press) 24 (22): 2493-8.

Sato, Kaoru, Yuka W. Iwasaki, Aoi Shibuya, Piero Carninci, Yuuta Tsuchizawa, Hirotsugu Ishizu, Mikiko C. Siomi, and Haruhiko Siomi. 2015. "Krimper Enforces an Antisense Bias on piRNA Pools by Binding AGO3 in the Drosophila Germline." *Molecular Cell* 59 (4): 553-563.

Saxe, Jonathan P, Mengjie Chen, Hongyu Zhao, and Haifan Lin. 2013. "Tdrkh is essential for spermatogenesis and participates in primary piRNA biogenesis in the germline." *The EMBO Journal* 32 (13): 1869-1885.

Sienski, Grzegorz, Derya Dönertas, Julius Brennecke, S.I. Grewal, V.L. Trudeau, M. Savitsky, A. Kalmykova, et al. 2012. "Transcriptional silencing of transposons by Piwi and maelstrom and its impact on chromatin state and gene expression." *Cell* (Elsevier) 151 (5): 964-80.

Sienski, G., J. Batki, K.-A. Senti, D. Dönertas, L. Tirian et al., 2015 Silencio/CG9754 connects the Piwi-piRNA complex to the cellular heterochromatin machinery. Genes Dev. 29: 2258–71.

Stump, A D, M Pombi, L Goeddel, J M C Ribeiro, J A Wilder, A della Torre, and N J Besansky. 2007. "Genetic exchange in 2La inversion heterokaryotypes of Anopheles gambiae." *Insect molecular biology* 16 (6): 703-9.

Van Rij, Ronald P., Maria Carla Saleh, Bassam Berry, Catherine Foo, Andrew Houk, Christophe Antoniewski, and Raul Andino. 2006. "The RNA silencing endonuclease Argonaute 2 mediates specific antiviral immunity in Drosophila melanogaster." *Genes and Development* 20 (21): 2985-2995.

Wallberg, Andreas, Fan Han, Gustaf Wellhagen, Bjørn Dahle, Masakado Kawata, Nizar Haddad, Zilá Luz Paulino Simões, et al. 2014. "A worldwide survey of genome sequence variation provides insight into the evolutionary history of the honeybee Apis mellifera." *Nature genetics* (Nature Publishing Group, a division of Macmillan Publishers Limited. All Rights Reserved.) 46 (10): 1081-8.

Wang, Xiao-Hong, Roghiyh Aliyari, Wan-Xiang Li, Hong-Wei Li, Kevin Kim, Richard Carthew, Peter Atkinson, and Shou-Wei Ding. 2006. "RNA Interference Directs Innate Immunity Against Viruses in Adult Drosophila." *Science* 312 (5772).

Weller, Andreas M, Christian Rödelsperger, Gabi Eberhardt, Ruxandra I Molnar, and Ralf J Sommer. 2014. "Opposing forces of A/T-biased mutations and G/C-biased gene conversions shape the genome of the nematode Pristionchus pacificus." *Genetics* 196 (4): 1145-52.

Whitfield, Charles W, Susanta K Behura, Stewart H Berlocher, Andrew G Clark, J Spencer Johnston, Walter S Sheppard, Deborah R Smith, Andrew V Suarez, Daniel Weaver, and Neil D Tsutsui. 2006. "Thrice out of Africa: ancient and recent expansions of the honey bee, Apis mellifera." *Science (New York, N.Y.)* 314 (5799): 642-5.

Xiol, Jordi, Pietro Spinelli, Maike A. Laussmann, David Homolka, Zhaolin Yang, Elisa Cora, Yohann Couté, et al. 2014. "RNA Clamping by Vasa Assembles a piRNA Amplifier Complex on Transposon Transcripts." *Cell* 157 (7): 1698-1711.

Yamamoto, Kimiko, Junko Nohata, Keiko Kadono-Okuda, Junko Narukawa, Motoe Sasanuma, Shun-Ichi Sasanuma, Hiroshi Minami, et al. 2008. "A BAC-based integrated linkage map of the silkworm Bombyx mori." *Genome biology* (BioMed Central Ltd) 9 (1): R21.

Yang, Z. 2007. "PAML 4: Phylogenetic Analysis by Maximum Likelihood." *Molecular Biology and Evolution* (Oxford University Press) 24 (8): 1586-1591.

Yang, Shao-Yu, Min-Jin Han, Li-Fang Kang, Zi-Wen Li, Yi-Hong Shen, and Ze Zhang. 2014. "Demographic history and gene flow during silkworm domestication." *BMC evolutionary biology* (BioMed Central Ltd) 14 (1): 185.

Yeom, Kyu-Hyeon, Yoontae Lee, Jinju Han, Mi Ra Suh, V. Narry Kim, Li Y., Hao Y.L., Ooi C.E., Godwin B., and Vitols E. 2006. "Characterization of DGCR8/Pasha, the essential cofactor for Drosha in primary miRNA processing." *Nucleic Acids Research* (Oxford University Press) 34 (16): 4622-4629.

Zamparini, Andrea L., Marie Y. Davis, Colin D. Malone, Eric Vieira, Jiri Zavadil, Ravi Sachidanandam, Gregory J. Hannon, and Ruth Lehmann. 2011. "Vreteno, a gonad-specific protein, is essential for germline development and primary piRNA biogenesis in Drosophila." *Development* 138 (18).

Zhang, Fan, Jie Wang, Jia Xu, Zhao Zhang, Birgit S. Koppetsch, Nadine Schultz, Thom Vreven, et al. 2012. "UAP56 Couples piRNA Clusters to the Perinuclear Transposon Silencing Machinery." *Cell* 151 (4): 871-884.

Zhang, Zhao, Jia Xu, Birgit S. Koppetsch, Jie Wang, Cindy Tipping, Shengmei Ma, Zhiping Weng, William E. Theurkauf, and Phillip D. Zamore. 2011. "Heterotypic piRNA Ping-Pong Requires Qin, a Protein with Both E3 Ligase and Tudor Domains." Molecular Cell 44 (4): 572-584.

Zhang, Z., J. Wang, N. Schultz, F. Zhang, S. S. Parhad et al., 2014 The HP1 Homolog Rhino Anchors a Nuclear Complex that Suppresses piRNA Precursor Splicing. Cell 157: 1353–1363.
