# Supplementary material for: RNA-Interference Pathways Display High Rates of Adaptive Protein Evolution in Multiple Invertebrates
Source: Genetics. 2018 Feb 1;208(4):1585–99. doi: 10.1534/genetics.117.300567 (PMC5887150; doi:10.1534/genetics.117.300567)
Supplement: Supplementary file 6 [file 1585FileS2.docx]

**Supplementary Figures**


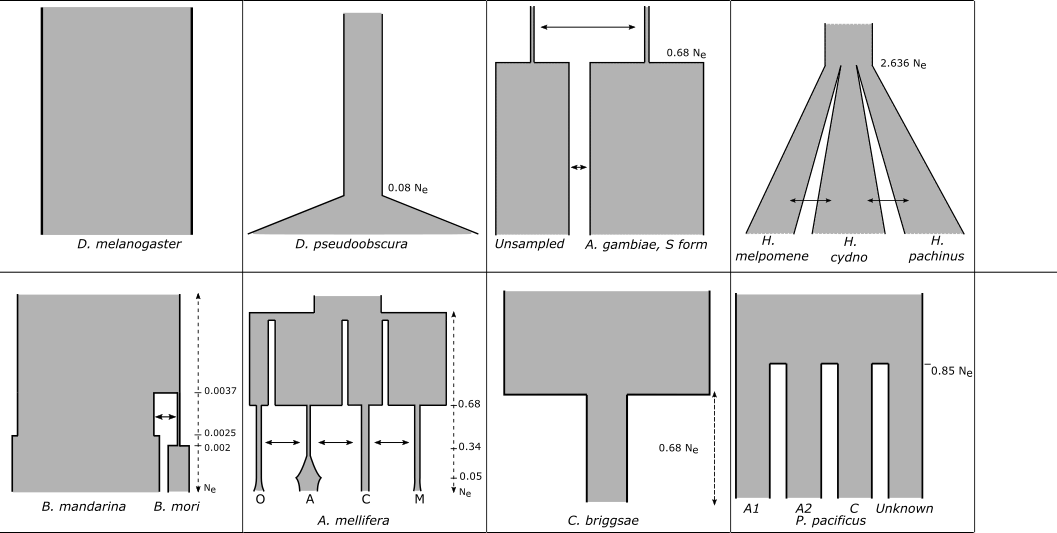


Figure S1 Demographic scenarios simulated for SweeD analysis

Coalescent simulations were performed using ms for demographic scenarios for each species which are supported by other studies. The African (Zambia) *D. melanogaster* were assumed to have a constant population size. *D. pseudoobscura* has recently undergone a population expansion 0.08 Ne generations ago. *A. gambiae* shares migrants with some other unknown, unsampled subpopulation which split 0.68 Ne generations ago. *Heliconius* species in Costa Rica split 2.636 Ne generations ago and have shared migrants since. *Bombyx mandarina* went through a small bottleneck when *B. mori* split, and shared migrants during that bottleneck (but not after). *Apis mellifera* have four subpopulations which have gone through multiple population expansions and bottlenecks, with all subpopulations sharing migrants until they join 0.68 Ne generations ago. *Caenorhabditis briggsae “*tropical samples” have undergone a population bottleneck 0.68 Ne generations ago. Finally, *Pristionchus pacificus* were sampled from four subpopulations, which split 0.85 Ne generations ago.


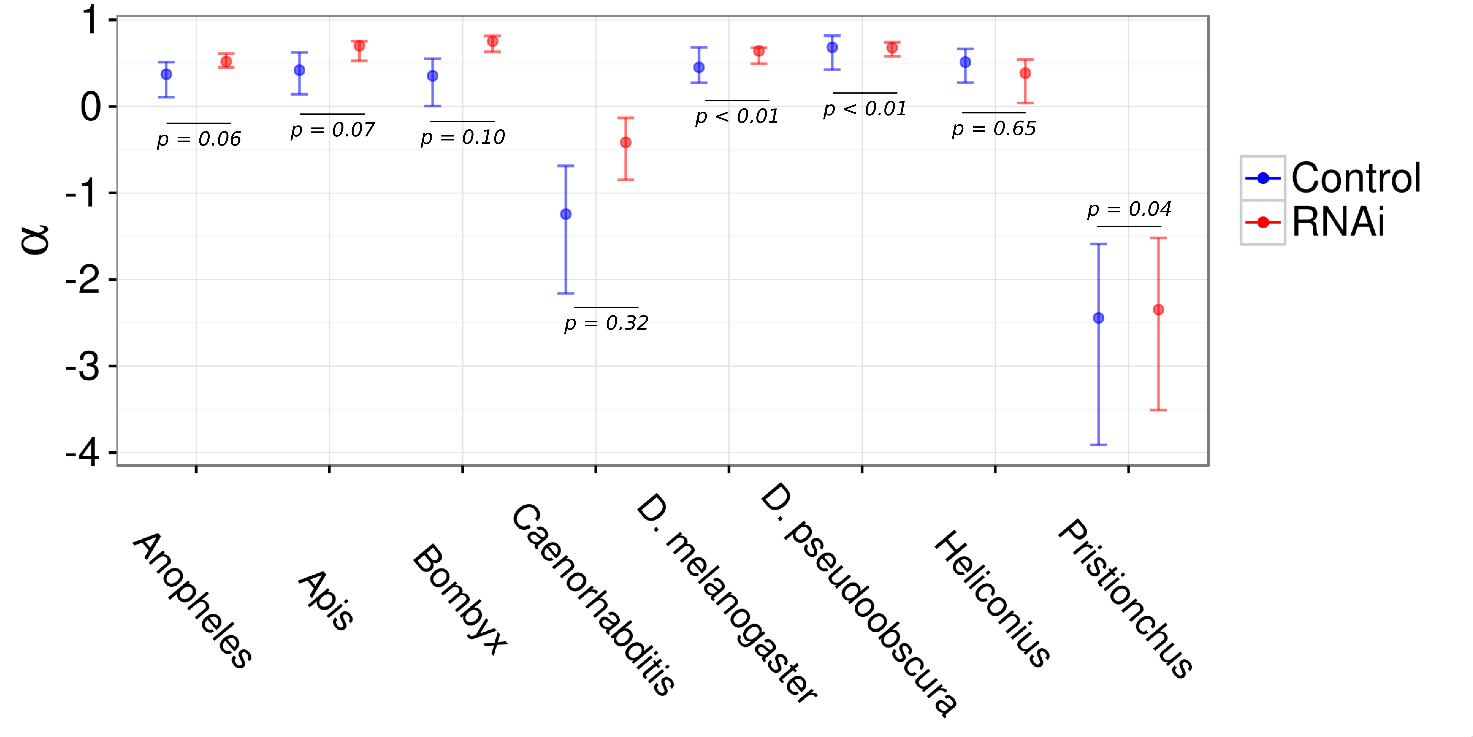


Figure S2: Alpha values for RNAi genes

For each species, α, or the proportion of adaptive substitutions was estimated from pooled polymorphism and divergence data using DFE-alpha for RNAi genes and position-matched control genes. α estimates for control genes are fairly constant across insect species, but are negative in the two nematode species. In all species except *H. melpomene*, the RNAi gene estimates are greater in RNAi genes than control genes.


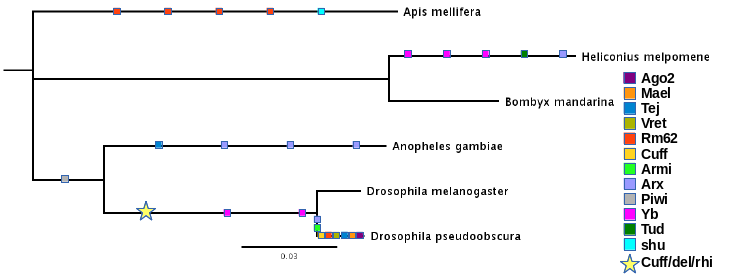


Figure S3 Possible duplications in RNAi pathway

Relationships of the insect species sampled, including coloured squares where possible gene duplications have occurred. Our search for RNAi genes in insect species other than *D. melanogaster* identified numerous duplications, and also some genes which were specific to *Drosophila*. Of note, *D. pseudoobscura* harboured duplications in *asterix, armitage, cutoff, rm62, vretano, tejas, maelstrom,* in addition to the multiple *AGO2* duplications reported previously (Lewis et al, 2016; Lewis et al, 2016), perhaps indicating an extensive addition to RNAi related pathways. *Asterix* was further duplicated three times in *Anopheles* and once in *Heliconius*, and *A. mellifera* also has five distinct copies of *rm62*. Furthermore, *yb* duplications have occurred independently in the lineage leading to *H. melpomene* and the one leading to the *Drosophila* species. The piRNA cluster transcriptional complex composed of cutoff, deadlock, and rhino were only observed in the two *Drosophila* species (represented by a star), and thus have likely either been lost in the other species or have evolved in since the split between *Anopheles* and *Drosophila*.


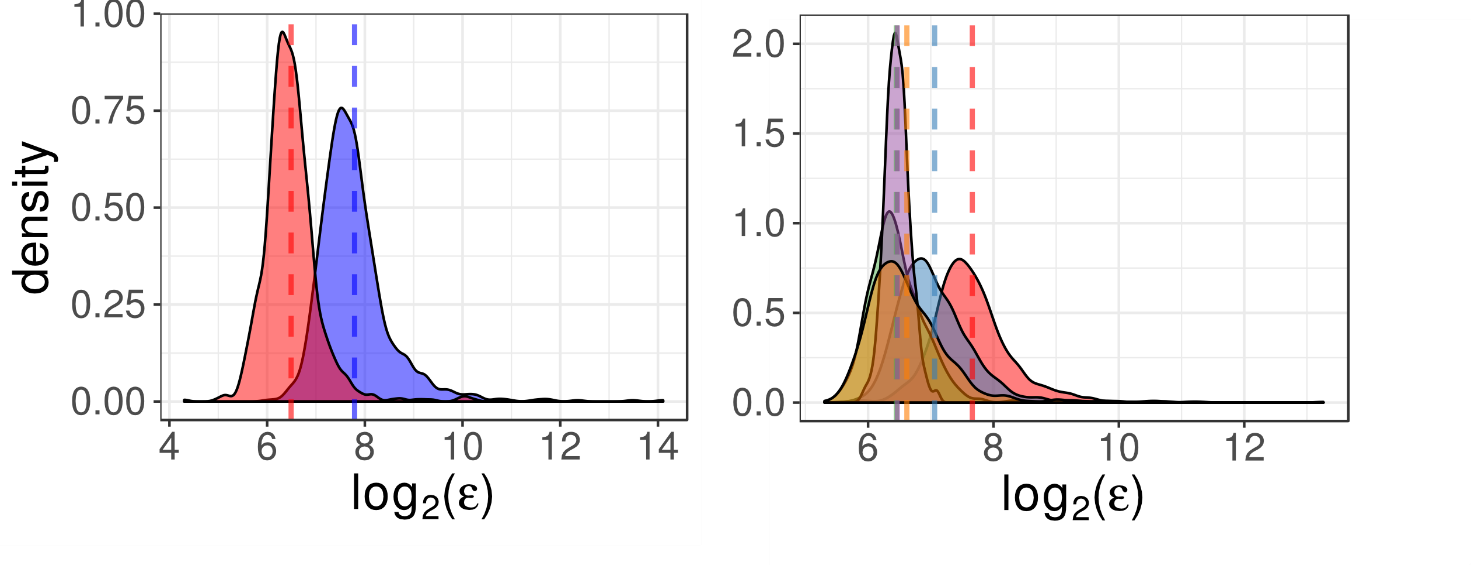


Figure S4 Coefficient of variation posterior distributions for residual variances in DFE-alpha meta-analysis

(Left) The coefficient of variation for the residual variance of RNAi genes (red) is not significantly greater than control genes (blue). (Right) This is also true for subpathway (piRNA: purple, viRNA: orange, miRNA: green, siRNA:blue) residual variances when combared with control genes (red). Therefore, the larger variances observed in rates of adaptive evolution in the RNAi pathway (Figure 1) and in RNAi subpathways (Figure 2) can be explained by an increase in the mean rates.


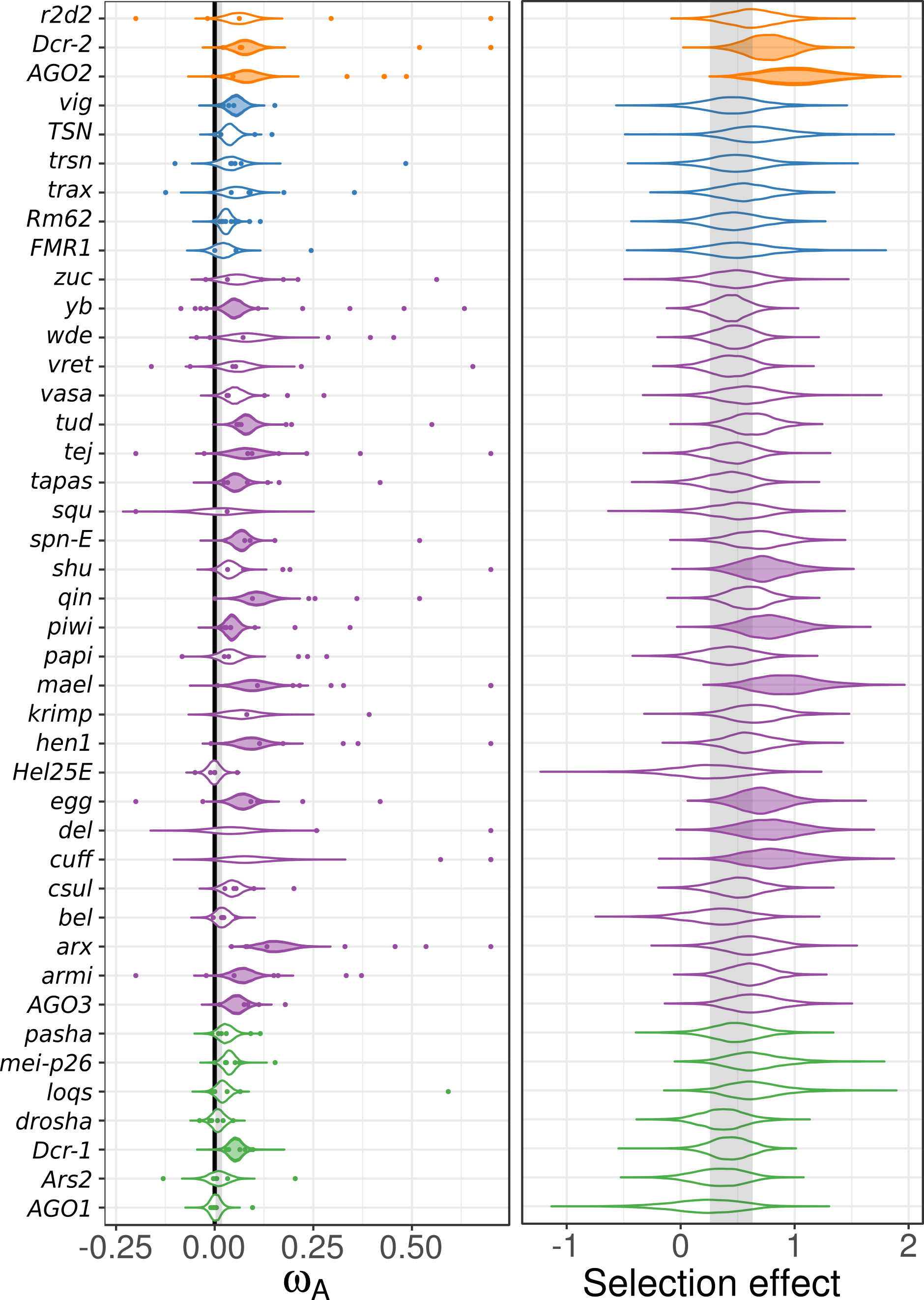


Figure S5 Cross-species homologue-level estimates of ω_A_ and selection effects without pathway assumptions

(Left) Individual gene ω_A_ estimates (coloured points) were calculated using DFE-alpha and analyses using a linear mixed model with species and gene as random effects (estimate uncertainty was included by incorporating bootstrap intervals as measurement error variance), but without subpathway as fixed effect (see Figure 4). The posterior distributions of the cross-species estimate for ω_A_ for each gene are plotted, and shaded if the MCMCp < 0.05 when tested against the control gene distribution (shaded grey region). Single-gene estimates of ω_A_ > 0.75 are plotted at 0.75 for clarity. (Right) The analogous analysis, except performed using SnIPRE, with the posterior distribution of homologue-level selection effects plotted. Both analyses find *AGO2, Dicer-2, piwi, maelstrom,* and *eggless* as having elevated protein substitution.


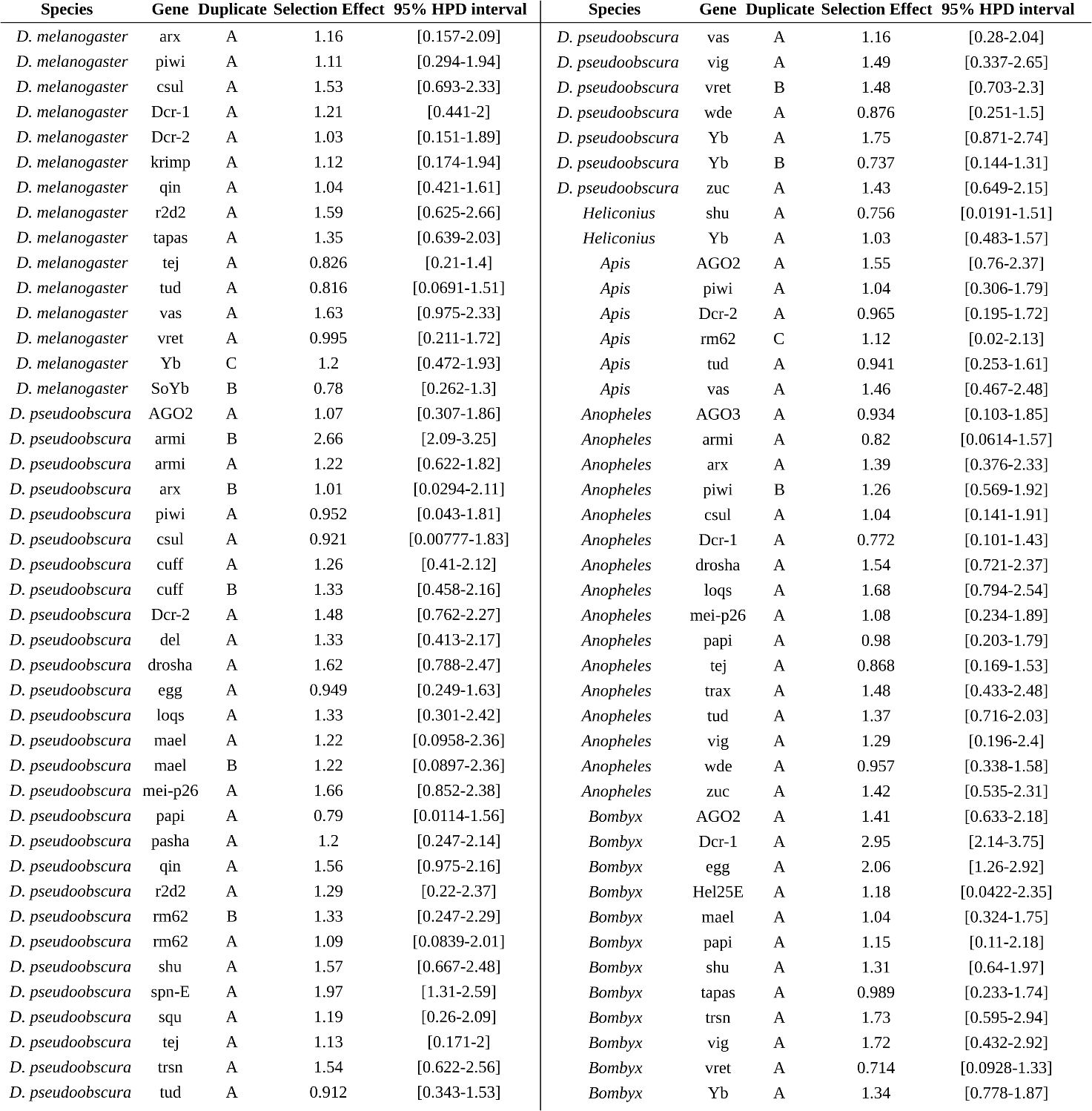


Figure S6 Genes with elevated selection effects

A list of RNAi genes with individually significant selection effects (i.e. a selection effect > 1) and their 95% highest posterior density intervals.


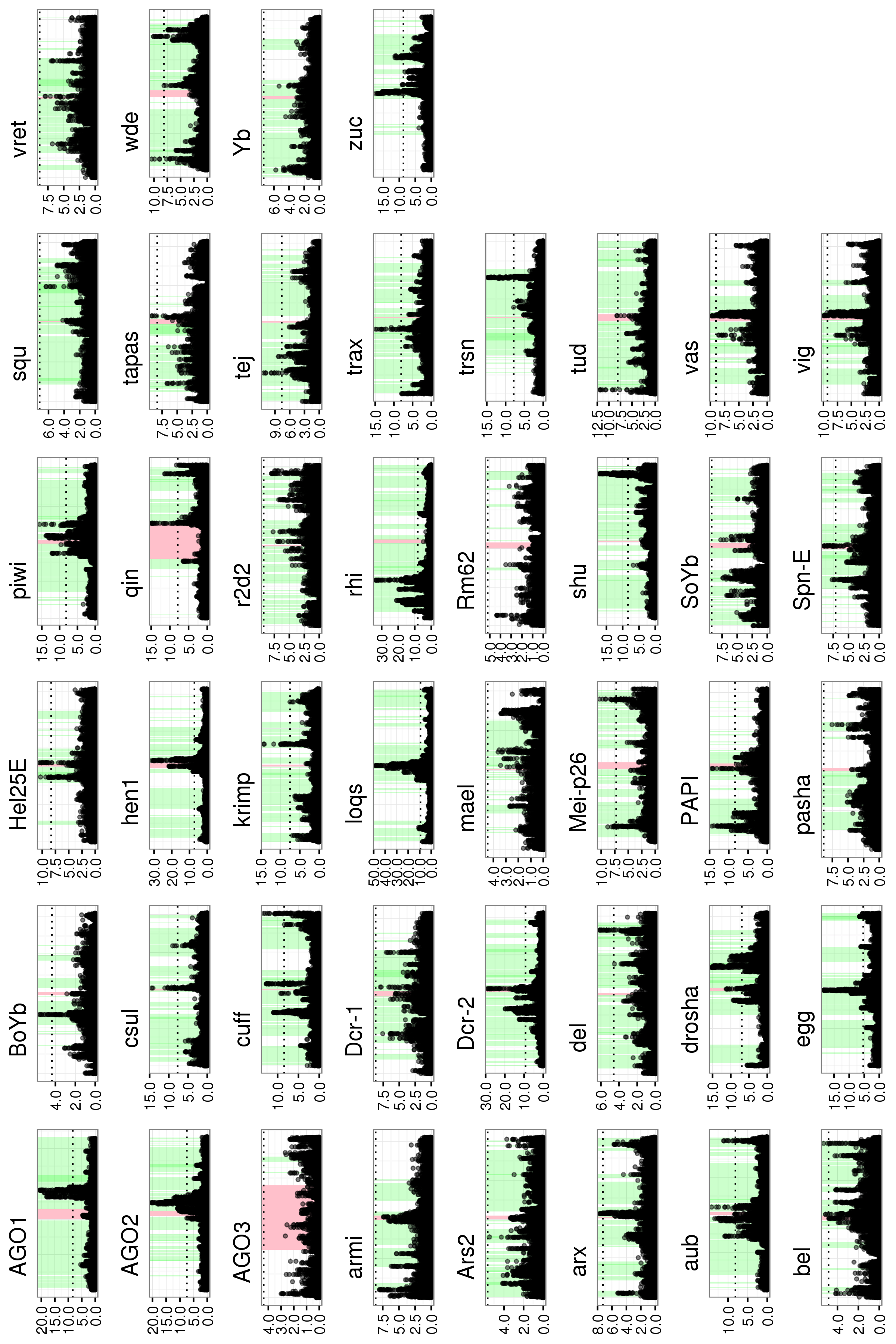


Figure S7: *Drosophila melanogaster* sweeps

For each *D. melanogaster* gene, the CLR statistic was plotted across a 200 kb region including the gene of interest. Each panel represents a region of the *D. melanogaster* genome, with red-shaded regions being the gene of interest and green-shaded regions being other genes along the chromosome. The horizontal dotted lines in each panel are significance thresholds calculated through neutral coalescent simulations.


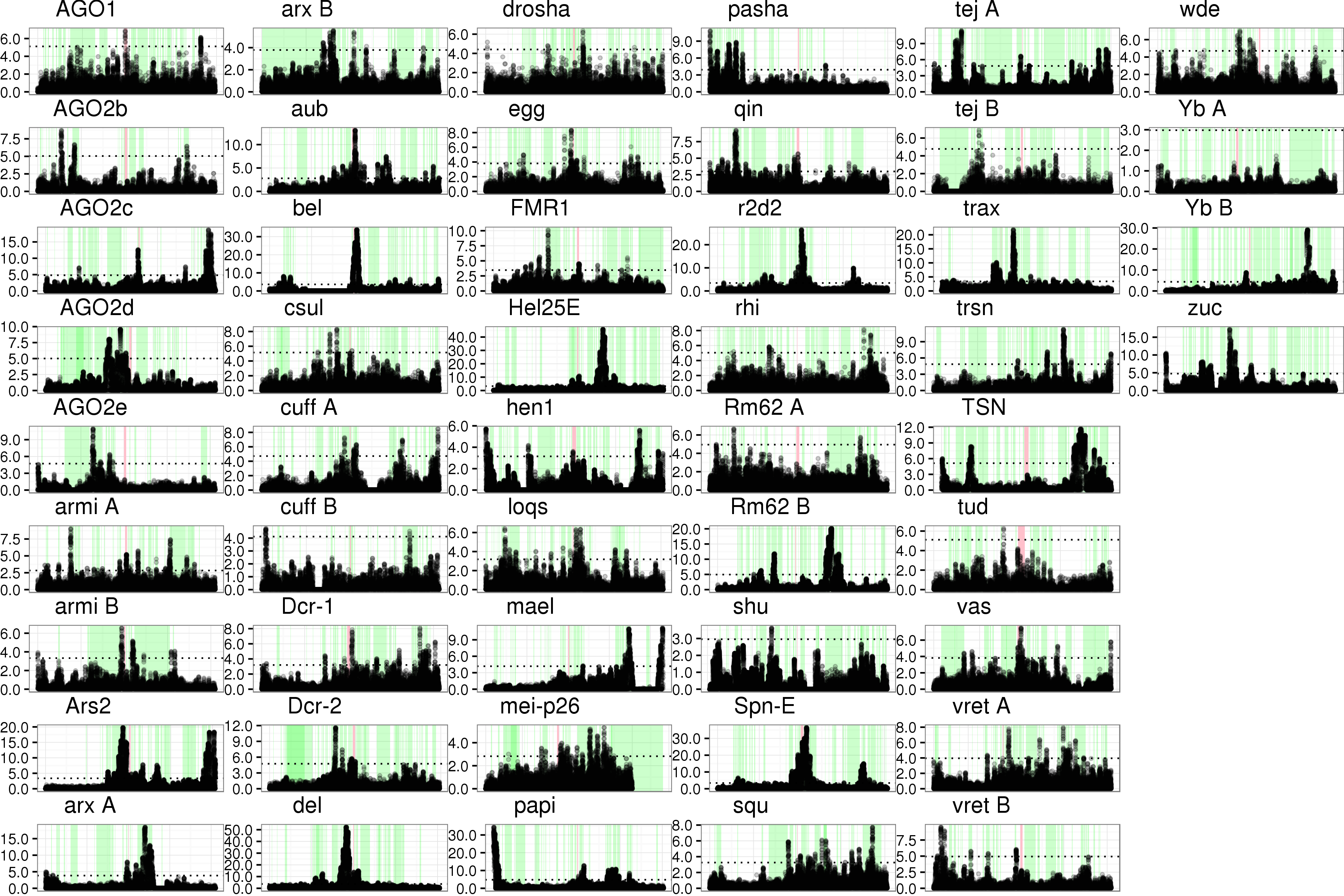


Figure S8: *Drosophila pseudoobscura* sweeps

For each *D. pseudoobscura* gene, the CLR statistic was plotted across a 200 kb region including the gene of interest. Each panel represents a region of the *D. pseudoobscura* genome, with red-shaded regions being the gene of interest and green-shaded regions being other genes along the chromosome. The horizontal dotted lines in each panel are significance thresholds calculated through neutral coalescent simulations.


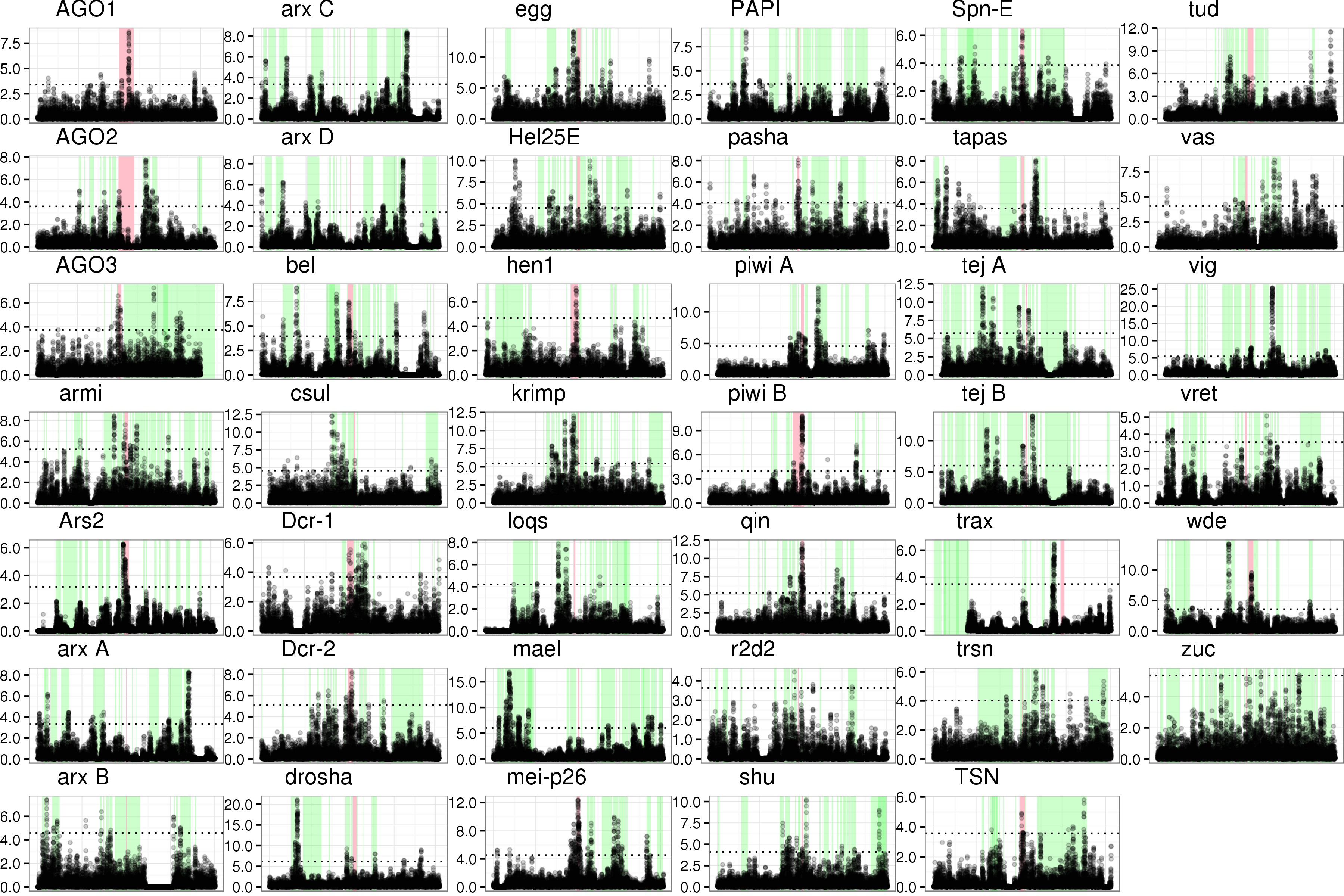


Figure S9: *Anopheles gambiae* sweeps

For each *A. gambiae* gene, the CLR statistic was plotted across a 200 kb region including the gene of interest. Each panel represents a region of the *A. gambiae* genome, with red-shaded regions being the gene of interest and green-shaded regions being other genes along the chromosome. The horizontal dotted lines in each panel are significance thresholds calculated through neutral coalescent simulations.


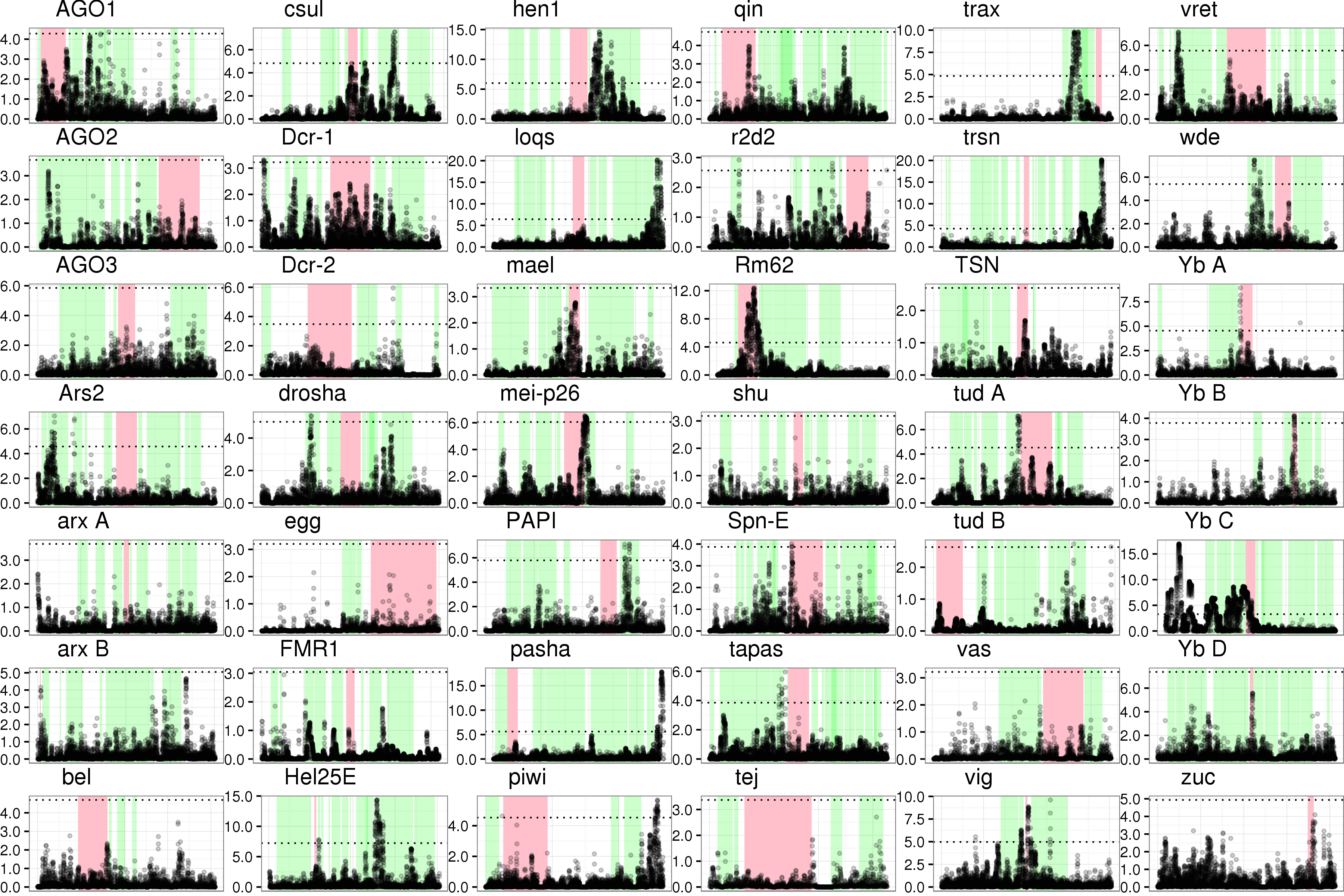


Figure S10: *Heliconius melpomene* sweeps

For each *H. melpomene* gene, the CLR statistic was plotted across a 200 kb region including the gene of interest. Each panel represents a region of the *H. melpomene* genome, with red-shaded regions being the gene of interest and green-shaded regions being other genes along the chromosome. The horizontal dotted lines in each panel are significance thresholds calculated through neutral coalescent simulations.


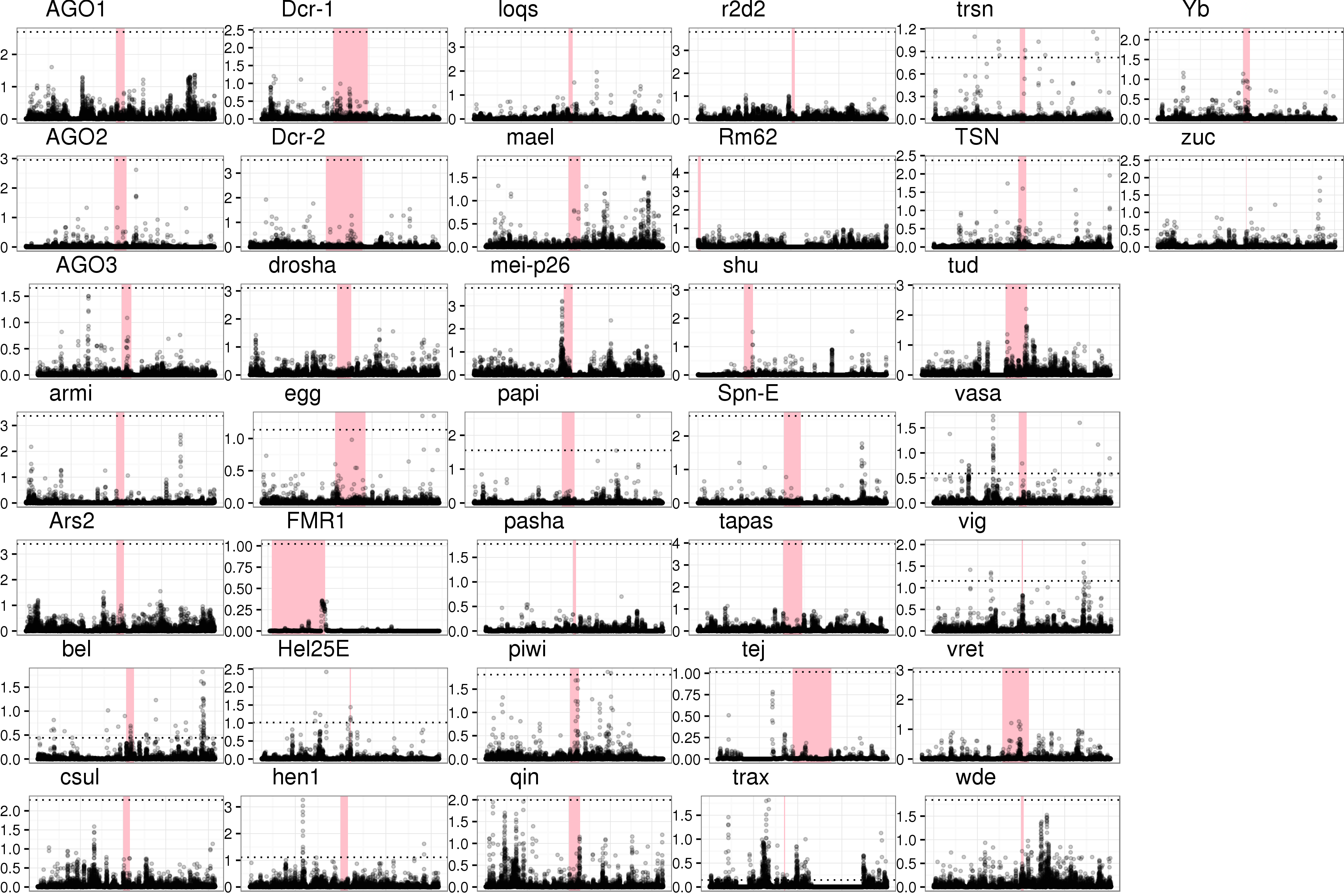


Figure S11: *Bombyx mandarina* sweeps

For each *B. mandarina* gene, the CLR statistic was plotted across a 200 kb region including the gene of interest. Each panel represents a region of the *B. mandarina* genome, with red-shaded regions being the gene of interest. The horizontal dotted lines in each panel are significance thresholds calculated through neutral coalescent simulations. The *Bombyx* genome used did not have an associated gff file, and so positions of nearby genes were not included.


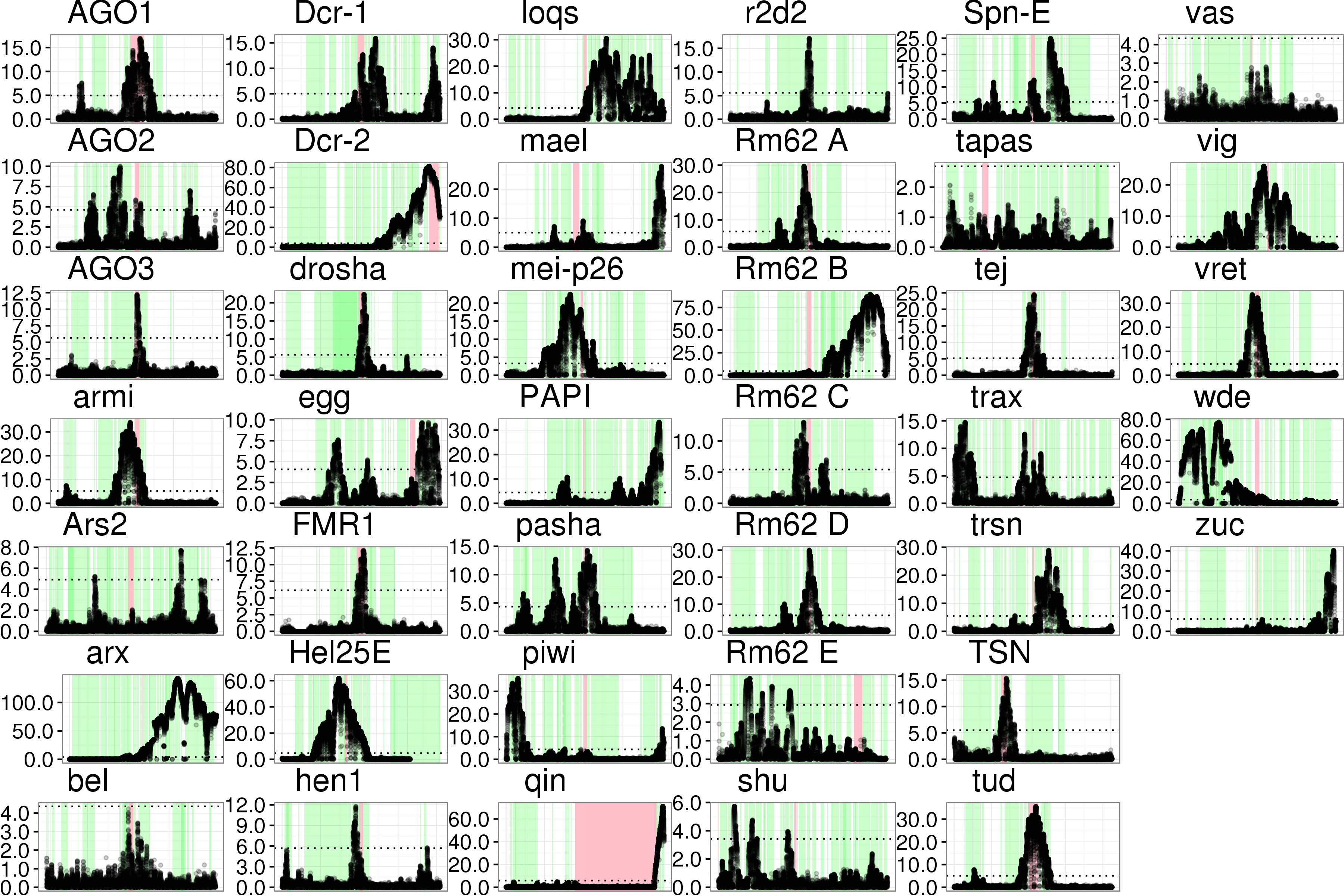


Figure S12: *Apis mellifera* sweeps

For each *A. mellifera* gene, the CLR statistic was plotted across a 200 kb region including the gene of interest. Each panel represents a region of the *A. mellifera* genome, with red-shaded regions being the gene of interest. The horizontal dotted lines in each panel are significance thresholds calculated through neutral coalescent simulations.


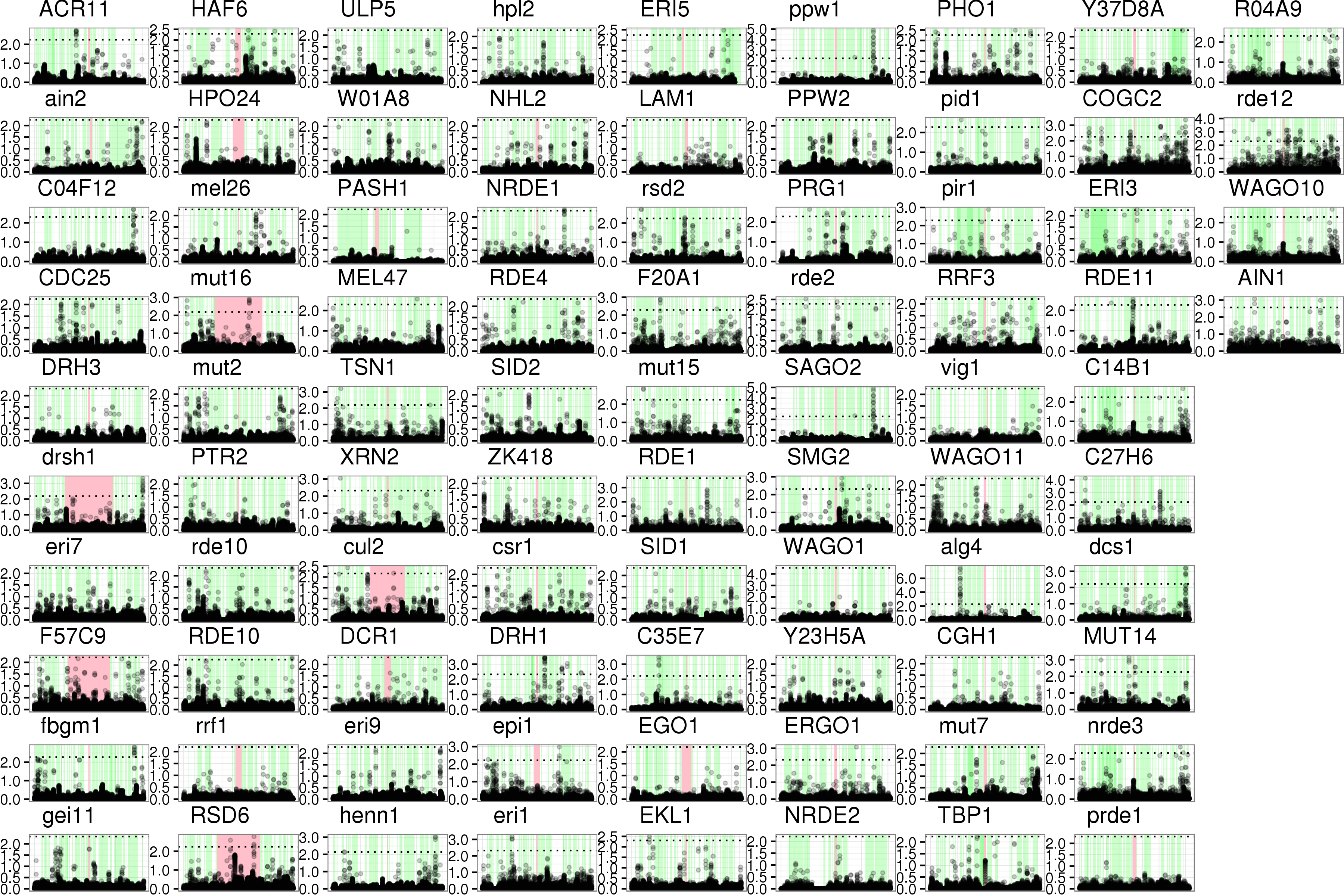


Figure S13: *Caenorhabditis briggsae* sweeps

For each *C. briggsae* gene, the CLR statistic was plotted across a 200 kb region including the gene of interest. Each panel represents a region of the *C. briggsae* genome, with red-shaded regions being the gene of interest. The horizontal dotted lines in each panel are significance thresholds calculated through neutral coalescent simulations.


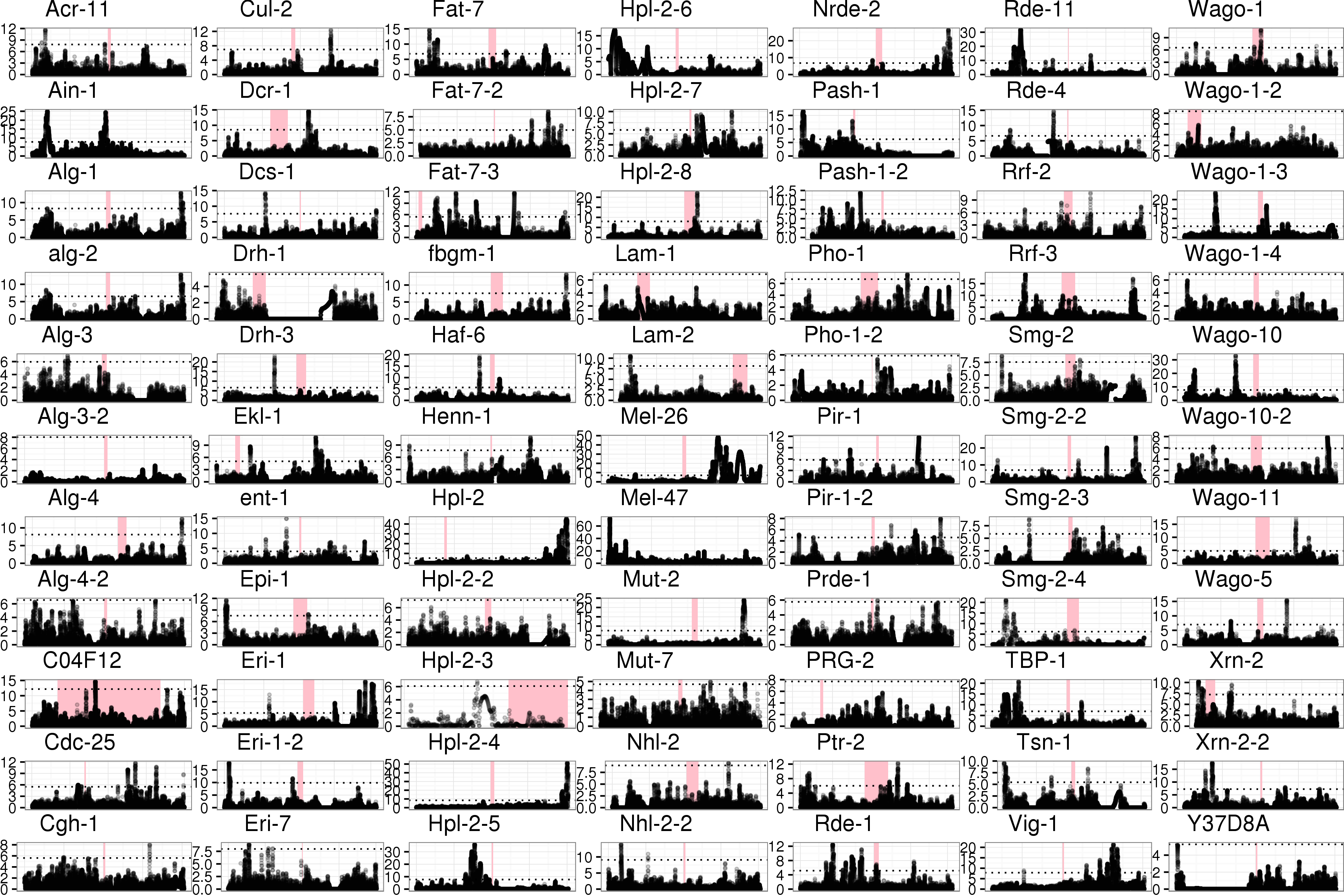


Figure S14: *Pristionchus pacificus* sweeps

For each *P. pacificus* gene, the CLR statistic was plotted across a 200 kb region including the gene of interest. Each panel represents a region of the *P. pacificus* genome, with red-shaded regions being the gene of interest. The horizontal dotted lines in each panel are significance thresholds calculated through neutral coalescent simulations. The *Pristionchus* genome used did not have an associated gff file, so positions of nearby genes were not included.


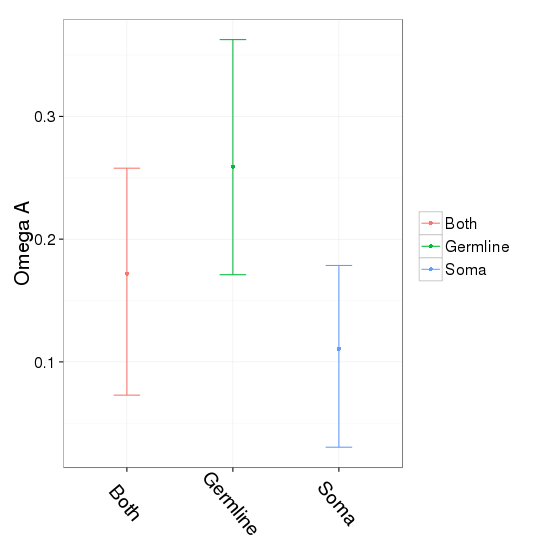


Figure S15: Germline and somatic piRNA pathway genes

Polymorphism and divergence from a larger set of piRNA pathway genes (those identified in two of three recent piRNA pathway screens, plus the core piRNA pathway) (Handler et al, 2013; Czech et al, 2013; Muerdter et al, 2013) in *D. melanogaster* were pooled based on whether they are active in the germline, soma, or both and used to calculate ω_A_. Confidence intervals were obtained by bootstrapping by gene 1000 times. Genes active in germline TE suppression show higher rates of adaptive protein evolution than those active in the somatic follicle cells, with genes active in both having an intermediate adaptive rate.

Data S1 Single-gene DFE-alpha meta-analysis input file

The output of DFE-alpha for each gene in each species, with sampling errors obtained by bootstrapping by codon.

Data S2 SnIPRE analysis input file

Raw polymorphism and divergence for nonsynonymous and synonymous sites in each gene in each species, used as input for the SnIPRE models.
